# Adolescent valuation of CARIES-QC-U: a child-centred preference-based measure of dental caries

**DOI:** 10.1186/s12955-022-01918-w

**Published:** 2022-02-03

**Authors:** H. J. Rogers, J. Sagabiel, Z. Marshman, H. D. Rodd, D. Rowen

**Affiliations:** 1 School of Dental Sciences, Faculty of Medical Sciences, Newcastle University, Newcastle-upon-Tyne, UK; 2 Department of Economics, Swedish University of Agricultural Economics, Uppsala, Sweden; 3 Unit of Oral Health, Dentistry and Society, School of Clinical Dentistry, University of Sheffield, Sheffield, UK; 4 Health Economics and Decision Science, School of Health and Related Research, University of Sheffield, Sheffield, UK

## Abstract

**Objectives:**

This study develops an adolescent value set for a child-centred dental caries-specific measure of oral health-related quality of life (OHRQoL) based upon CARIES-QC (Caries Impacts and Experiences Questionnaire for Children). This study develops a new approach to valuing child health by eliciting adolescent preferences and anchoring these onto the 1–0 full health-dead QALY (quality adjusted life year) scale using ordinal adult preferences.

**Methods:**

Two online surveys were created to elicit preferences for the CARIES-QC classification system. The first comprised best–worst scaling (BWS) tasks for completion by adolescents aged 11–16 years. The second comprised discrete choice experiment tasks with a duration attribute (DCE_TTO_) for completion by adults aged over 18 years. Preferences were modelled using the conditional logit model. Mapping regressions anchored the adolescent BWS data onto the QALY scale using adult DCE_TTO_ values, since the BWS survey data alone cannot generate anchored values.

**Results:**

723 adolescents completed the BWS survey and 626 adults completed the DCE_TTO_ survey. The samples were representative of UK adolescent and adult populations. Fully consistent and robust models were produced for both BWS and DCE_TTO_ data. BWS preferences were mapped onto DCE_TTO_ values, resulting utility estimates for each health state defined by the classification system.

**Conclusion:**

This is the first measure with predetermined scoring based on preferences to be developed specifically for use in child oral health research, and uses a novel technique to generate a value set using adolescent preferences. The estimates can be used to generate QALYs in economic evaluations of interventions to improve children’s oral health.

**Supplementary Information:**

The online version contains supplementary material available at 10.1186/s12955-022-01918-w.

## Introduction

Dental caries, also known as tooth decay, is the most common chronic condition to affect children globally. It causes significant negative impacts on the lives of children and young people, including pain, local infection and in some cases may lead to emergency hospitalisation due to spread of the infection and systemic illness. In the UK, dental caries remains the most common reason for children to require a general anaesthetic, with an estimated annual cost of £39 million (approximately 52447000 US Dollars) to the National Health Service [1].

Dental caries is a largely preventable disease, thus there are a range of different programmes available to reduce the prevalence in children. However, there have been few economic evaluations to determine the cost effectiveness of such programmes. Within child oral health research, this paucity of economic evaluations could be attributed to the lack of a suitable instrument to measure Quality Adjusted Life Years (QALYs). QALYs measure the benefit of a healthcare intervention, combining the quality and length of life gained to produce a single index. The quality of life component is represented in terms of utilities, which reflect an individual’s preferences for different health states. The Child Health Utility-9 Dimensions (CHU9D), a generic paediatric preference-based measure (PBM) has been shown to lack sensitivity to changes in caries status [2]. The lack of use of other measures and the poor psychometric performance of CHU9D suggests that the content of child and adolescent generic PBMs may not be appropriate or sensitive for use in oral health research [3]. There is an established need for the development of a validated PBM, specifically for children, that is appropriate for measuring treatment benefits for dental caries [2–4]. This was considered achievable through the adaptation of a novel child-centred caries-specific oral health-related quality of life (OHRQoL) measure, known as CARIES-QC (Caries Impacts and Experiences Questionnaire for Children) [5]. CARIES-QC was developed with involvement of children at every stage and has been validated for use with 5–16 year olds [5].

The decision around whose preferences should be used to generate utility values for child and adolescent-specific PBMs is a normative decision, with no clear guidelines from international agencies around whose preferences should be elicited to inform policy. Adolescent preferences can be argued for on the grounds that children and adolescents experience the health states and therefore it is their preferences that are most relevant [6, 7]. The use of ordinal techniques, such as discrete choice experiments (DCE) and best–worst scaling (BWS), have shown promise as methods to access children and young people’s preferences [8–10]. These offer a number of key advantages over cardinal tasks, such as standard gamble (SG) and time trade-off (TTO). Cardinal tasks are considered to be particularly cognitively demanding, and require respondents to consider the risk of death, or trading years of their life respectively; features that raise ethical concerns when used with children and young people [11]. Pairwise DCE tasks require the respondent to state their preference between two hypothetical health states, each with described characteristics, whilst the most commonly employed variant of the BWS method, known as BWS Case 2 task, presents the respondent with one health state profile and asks them to choose the best feature and the worst feature [12]. Through repeating this process numerous times with varying attribute level combinations, preference weights can be estimated. Whilst both types of task have been used effectively to gain preference weights from younger populations, emerging evidence suggests adolescents have a greater understanding of BWS tasks, compared to DCE [9, 10, [13, 14]. Whilst neither of these tasks allow values to be anchored onto the 1–0 full health to dead QALY scale, methods to overcome this have been described in the literature [15]. One such way, is to re-scale modelled preferences obtained using BWS using preferences elicited via a cardinal approach, such as TTO, or DCE_TTO_ which is DCE with a duration attribute that enables modelled preferences to be anchored directly onto the 1–0 full health-dead scale [16–19]. However, as these cardinal approaches may be unsuitable for children and young people for the reasons described earlier, the re-scaling values may need to be obtained from adults; an approach used to generate adolescent value sets for the Child Health Utility 9 Dimension (CHU9D) instrument in Australia and China [16, 17].

This paper describes the adolescent valuation of a classification system for a PBM based upon CARIES-QC, to enable this measure to be used to generate QALYs for use in cost-effectiveness analyses. This comprises three stages: (1) A BWS survey completed by an adolescent sample, modelled using regression analyses to generate latent utility values; (2) A concurrent DCE_TTO_ survey in adults, modelled to generate utility values anchored on the 1–0 full-health-dead scale; (3) Mapping of the adolescent modelled BWS latent utility values onto the DCE_TTO_ modelled utility values to generate adolescent utility values that are on the 1–0 full-health dead scale required to generate QALYs. For the purposes of this paper, the term ‘child’ refers to those aged 5–16 years, and ‘adolescent’ refers to those aged 11–16 years.

## Methods

Ethical approval for this study was provided by Yorkshire and the Humber Research Ethics Committee (18/YH/0148).

### Classification system

CARIES-QC is a unidimensional measure containing 13 questions (Additional file 1: Table S1), each with three response options relating to severity (*‘not at all’*, *‘a bit’* and *‘a lot’*) that were identified by children during its development. The decision to select CARIES-QC as the basis from which to derive a classification system for this PBM was taken after a critical review of alternative measures of paediatric OHRQoL, of which many were not developed specifically to capture the impacts of caries and hence may lack the psychometric properties to detect changes in caries status arising from an intervention [20]. Moreover, few of these measures have involved children in their development, and hence may not reflect the views of the relevant population [5, 20]. As it was developed in a UK setting, the features of CARIES-QC were considered directly applicable to the population in the present study. The psychometric properties of CARIES-QC are also favourable; it has been shown to have good face and content validity (determined using a child-centred qualitative approach), construct validity (demonstrated by strong statistically significant correlations with clinical data: *p* < 0.01), responsiveness (reduction in mean scores between baseline and follow-up for children who felt they had improved: − 4.42, SD:3.62) and reliability (Cronbach’s alpha: 0.9) [5, 21].

The identification of attributes for a classification system (Table 1) for a PBM from CARIES-QC consisted of Rasch analysis, classical psychometric testing, involvement of child and parent representatives, and involvement of the developers of CARIES-QC. The details of this process are described elsewhere [22].

**Table 1:** The CARIES-QC-U classification system

Dimensions	Level	Variables	Health state descriptors
Hurt	1	Hurt1	My teeth do not hurt me at all
	2	Hurt2	My teeth hurt me a bit
	3	Hurt3	My teeth hurt me a lot
Annoy	1	Annoy1	My teeth do not annoy me at all
	2	Annoy2	My teeth annoy me a bit
	3	Annoy3	My teeth annoy me a lot
Kept awake	1	Awake1	My teeth do not keep me awake at all
	2	Awake2	My teeth keep me awake a bit
	3	Awake3	My teeth keep me awake a lot
Hard to eat	1	Eat1	My teeth do not make it hard to eat some foods
	2	Eat2	My teeth make it a bit hard to eat some foods
	3	Eat3	My teeth make it really hard to eat some foods
Cried	1	Cry1	My teeth do not make me cry at all
	2	Cry2	My teeth make me cry a bit
	3	Cry3	My teeth make me cry a lot

Duration* in life years	Soft launch variable	Main survey variable	
	LY1 (1 year)	LY6m (6 months)	
	LY4 (4 years)	LY12m (1 year)	
	LY7 (7 years)	LY18m (1 year 6 months)	
	LY10 (10 years)	LY24m (2 years)	

Reproduced from Rogers et al., 2020

*Attribute included in the DCE_TTO_ survey only

### Preference elicitation technique

BWS tasks were used to elicit preferences from an adolescent population, as previous research conducted by the authors using the CARIES-QC-U classification system suggests that adolescents find these tasks easier to understand than DCE [14].

### Anchoring adolescent preferences onto 1–0 full-health dead scale

DCE_TTO_ was selected to obtain cardinal utilities from adults that could be used to map adolescent scores onto the 1–0 QALY scale. This is a novel application of DCE_TTO_ that has not to our knowledge been used previously for this purpose despite the wealth of evidence demonstrating successful delivery of DCE_TTO_ in the format of an online survey, allowing the researcher to collect data rapidly for a large sample [23, 24]. The use of DCE_TTO_ also provided the flexibility of enabling a value set based on adult preferences to be generated.

In-keeping with the literature, the DCE_TTO_ duration attributes included four levels; one year, four years, seven years and ten years [25–28]. Each health state within the DCE_TTO_ was simply labelled A or B, to prevent potential heuristics due to label content [29]. Respondents were asked which health profile they would prefer for themselves and were not aware that the health states were child or adolescent health states (for a discussion of the advantages and disadvantages of this approach see Rowen et al. [11]).

### Selection of health state profiles for valuation

Prior to valuation, it is necessary to select the health state profiles to be valued. Prior analysis of CARIES-QC-U confirmed that the dimensions do not co-occur and it is therefore appropriate to use approaches for selecting health state profiles for valuation that assume independence between the dimensions.

#### BWS

A full factorial design was used for the BWS survey, comprising all 243 health states, so that every health state was valued directly. The same approach was used in a study eliciting adolescent preferences for EQ-5D-Y using BWS tasks, a generic paediatric PBM that has the same number of attributes and levels in each attribute [30]. For each respondent, health states were randomly selected from all 243 possible health states. This ensured that each health state was valued an approximately equal number of times.

#### DCE_TTO_

To minimise participant fatigue (and resultant errors), and maximise completion rates, each participant was presented with nine tasks. To allow for this, the number of choice tasks chosen was 120 (which is far greater than the number of coefficients to estimate) and the design was blocked into groups of nine. The design was generated using the d-create add-in using Stata (StataCorp LLC, Texas, USA) [31]. The d-create command generates a D-efficient design and uses the modified Federov algorithm [32–35].

### Survey design

Two surveys were developed with input from child and adult patient and public involvement (PPI) representatives. The surveys were intended to be as similar as possible, with the obvious exception of the task itself, to minimise any differences surrounding the context in which the tasks were completed. Colour scheme and font for both surveys was chosen in accordance with national guidance to aid participants with specific learning difficulties and visual impairments to improve accessibility [36].

Participants were presented with basic sociodemographic questions surrounding age, gender and ethnicity. Postcodes were requested to determine the geographical spread of participants amongst the devolved nations of England, Wales, Scotland and Northern Ireland, and also to determine levels of deprivation in accordance with the most recent Indices of Multiple Deprivation tools for the respective nations [37–40]. The adult survey also enquired into participants’ marital and employment status to determine whether the sample was nationally representative for these factors.

Following this, participants were asked to complete a series of questions regarding their general health, dental health and previous experience of caries, alongside the questions used to form the CARIES-QC-U classification system that were intended to familiarise participants with the wording used in the tasks, and to engage them in thinking about teeth.

Basic information about tooth decay was provided in these surveys, accompanied by an appropriate photograph of a decayed tooth, as chosen by adolescent PPI representatives.

**Fig. 1:**
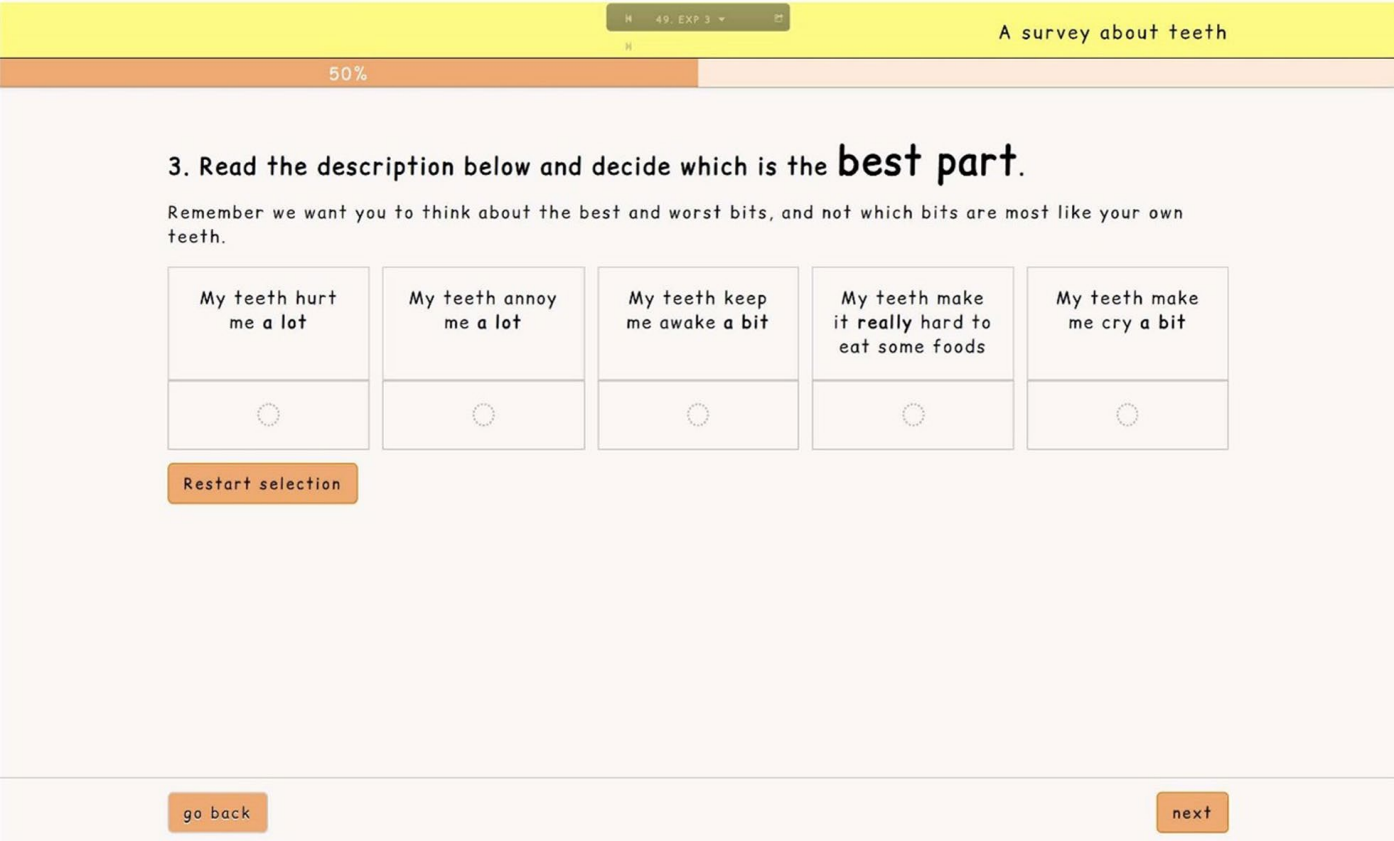
Screenshot of the BWS survey for adolescent participants

**Fig. 2:**
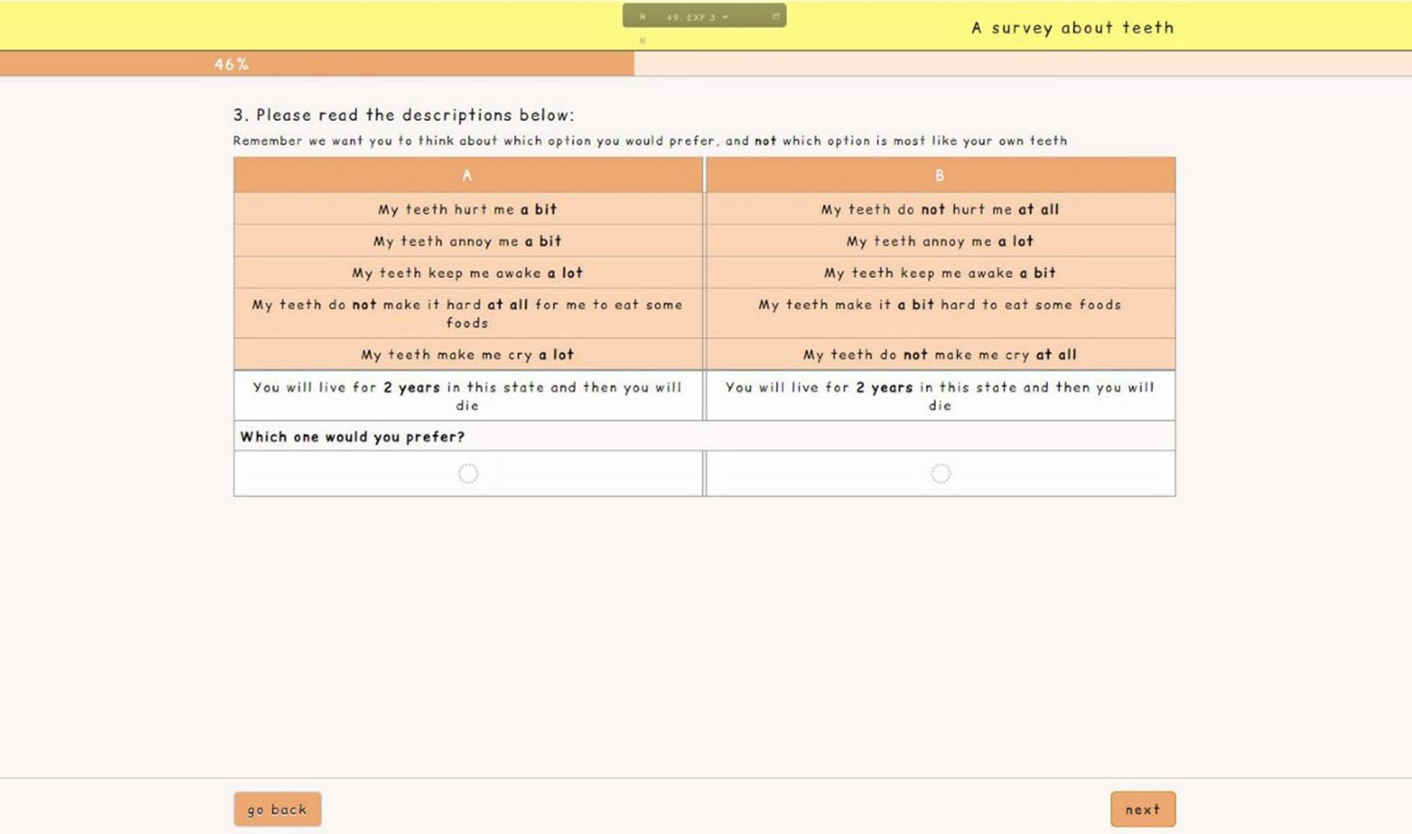
Screenshot of the DCE_TTO_ survey for adult participants

In line with previous research by the authors, adolescent respondents were then allocated eight BWS tasks (Fig. 1) to complete [14], whilst adult participants were allocated nine DCE_TTO_ tasks (Fig. 2). A ‘walkthrough’ was incorporated into each survey, to demonstrate how the task should be answered, followed by a practice question. For the adult survey, this practice question also acted as a dominance test.

The surveys both concluded with two summary questions, regarding the participants’ difficulty of understanding the tasks and difficulty of making a choice. Three response options were provided following the recommendations of adolescent PPI representatives (‘easy’, ‘difficult’ and ‘somewhere in the middle’).

### The sample

Surveys were hosted by SurveyEngine (SurveyEngine GmbH, Berlin, Germany) and promoted on a number of online survey platforms across the United Kingdom (UK). Participation was voluntary, with nominal incentives provided by the survey platforms in accordance with their standard procedures.

A total of 700 participants was considered to be sufficient to produce stable data for each survey and would facilitate a soft launch to be conducted to allow the data to be reviewed and any necessary alterations to be made to the survey before completion by the remaining participants [24, 41]. As such, a sample size of 700 adolescent participants and 700 adult participants were recruited for the surveys. The first 100 adolescents and 100 adults that were recruited formed the sample for the soft launch, whilst the remaining 600 adolescents and 600 adults formed the main survey sample. A sample size of 600 for each main survey enables each state to be seen on average 20 times in the BWS survey, and for each choice set to be seen on average 45 times in the DCE_TTO_ survey.

Survey platform members aged over 18 years were invited to participate in the adult survey, whilst children of members were invited to complete the adolescent survey providing they were aged between 11 and 16 years. Those who were happy to take part were asked to consent and assent respectively; parental consent was also required for adolescent participants. Quotas were set for age to ensure a representative sample was obtained for each survey.

All data obtained from the soft launch were analysed first to identify any discrepancies, and to ensure that the surveys were functioning as intended. Any required changes to the surveys were then made before the main surveys were launched.

### Analysis

Sociodemographic data were analysed using descriptive statistics to determine the diversity of the samples, which were compared against data from the UK 2011 Census [42]. Postcode data were used to determine geographical spread of participants across the devolved nations, and the level of deprivation of the area in which participants’ resided using the relevant Index of Multiple Deprivation tool [37–40]. Self-reported oral health outcomes, difficulty of understanding and difficulty of choice were also analysed descriptively.

Marginal choice frequencies were determined for the BWS survey data by dividing the number of times a domain level was selected as being ‘best’ or ‘worst’, by the number of times that domain level was available to be chosen within the survey [30].

The proportion of participants passing the DCE_TTO_ dominance test was determined. There is no accepted dominance test for BWS.

#### Modelling BWS data

Values for the BWS data were estimated in Stata/MP 16.0 (StataCorp LLC, Texas, USA) using conditional logistic regression in line with previous research [13, 41, 43].

The equation to be estimated was specified as:

$$U_{id} = X_{dl} \beta_{dl}^{\prime } + \varepsilon_{dl}$$

where $U_{id}$ represents the utility that individual *i* derives from choosing dimension *d* and $X_{dl}$ represents a vector of CARIES-QC-U attribute levels where *d* represents the 5 dimensions and *l* = 1,2,3 represents the 3 severity levels, $\beta_{dl}^{\prime }$ is the vector of coefficients where, for example, $\beta_{11}$ represents the coefficient for attribute 1 (hurt) level 1 (‘not at all’) and $\varepsilon_{dl}$ is the random component [44].

The conditional logit model considers all choice options as attribute levels, rather than complete states. The ‘worst’ choice data can then be appended to the ‘best’ choice data for each health state scenario, to form a best–worst pair [43, 45]. The model then generates attribute level values on a latent scale (note this is not anchored on the 1–0 full health-dead scale required to generate QALYs) [41]. The dimension with the highest marginal frequency for ‘best’ at level 1 (‘not at all’) was selected as the reference for the model.

The sign and magnitude of the coefficients were reviewed for significance at the 5% level, as well as logical consistency, whereby there is an expectation that the utility value decreases (or at least stays the same) as OHRQoL deteriorates [27]. Any inconsistent adjacent levels were merged to produce a single utility decrement that can be applied when the dimension is at either of the respective levels, in line with previous studies of this type, before re-estimating a fully consistent model [27].

The reduced sample approach was used to explore the presence and impact of heterogeneity relating to gender, age, self-reported general and dental health, and previous caries experience.

#### Modelling DCE_TTO_ data

Regressions were estimated for the DCE_TTO_ data using the conditional logit model, in line with previous studies [18, 46]. The formula for this model is:

$$\mu_{ij} = \alpha_{i} + \beta_{1} t_{ij} + \beta_{2}^{\prime } {\mathbf{x}}_{ij} t_{ij} + \varepsilon_{ij}$$

where $\mu_{ij}$ represents the utility of individual *I* for health state profile *j*, ${ }\alpha_{i}$ is an individual specific constant term, $\varepsilon_{ij}$ represents the error term, $\beta_{1}$ is the coefficient for duration in life years *t* and $\beta_{2}^{\prime }$ represents the coefficients on the 10 interaction terms of duration and attribute variables composed of levels 2 and 3 of each quality of life attribute (where level one is the baseline).

The values were converted from being on a latent scale, to the 1–0 full health-dead scale using the marginal rate of substitution [18], whereby each coefficient is divided by the coefficient for duration: $\frac{{\beta }_{2ij}}{{\beta }_{1}}$. This produces a utility weight for each level of each dimension. The sum of the utility weights for the relevant level of each dimension can be added to 1 in order to generate anchored utility values on the 1–0 full health-dead scale [18].

As with the BWS, the sign and magnitude of the coefficients were reviewed for significance at the 5% level, as well as logical consistency [27]. Any inconsistent adjacent levels were merged to produce a single utility decrement that can be applied when the dimension is at either of the respective levels, in line with previous studies of this type [27]. The fully consistent model was then estimated.

The duration attribute was modelled as a linear and continuous variable, hence it was necessary to confirm this assumption was correct through conducting a test of linearity [26, 47]. Duration was modelled as a categorical variable and the coefficients plotted.

Interaction terms were included to explore the presence and impact of heterogeneity with regard to gender, age, employment, marital status, self-reported general and dental health, and previous caries experience. The unanchored coefficients were then reviewed to determine the impact of these different characteristics on the results. Positive interactions indicate that there is a lower utility decrement to the attribute level, so the overall utility values for health states are higher (closer to 1). This approach was used rather than a reduced sample approach since it easily enabled the impact to be determined across a large number of different characteristics, in particular where there were not a large number of respondents. The reduced sample approach, as applied to the BWS data, was also examined and found the same results.

#### Model robustness

In order to determine robustness, models were re-estimated using a reduced sample approach to exclude participants who failed the dominance test (DCE_TTO_ data only), those who found it difficult to choose an answer, those who found the tasks difficult to understand, participants who completed the survey very quickly, and those who took a long time to complete it [18]. The authors reviewed the extent to which the coefficients were affected by the exclusion of these participants and a decision was made on whether to proceed with or without these participants [18, 48].

#### Anchoring adolescent BWS values onto the QALY scale

The mapping approach was used to estimate cardinal values (DCE_TTO_) for the latent BWS values for all health states:

$${\text{DCETTO}}_{j} = f\left( {{\text{BWS}}_{j} } \right) + \varepsilon_{j}$$

where DCETTO_*j*_ represents the mean modelled DCE_TTO_ utility of health state *j*, BWS represented the modelled latent utility value for health state *j*, and ${\varepsilon }_{j}$ is the error term [15]. This assumes a linear approach with an intercept.

Mean modelled DCE_TTO_ utility values and BWS latent utility values for all health states were plotted and reviewed for linearity. Ordinary least squares regressions were estimated to generate the mapping models mapping the BWS latent values onto DCE_TTO_ values [15]. The inclusion of squared and cubic terms were explored to determine the most appropriate model specification [15]. The mapped utility predictions were then plotted and compared to the modelled BWS latent values and DCE_TTO_ values.

All modelling was undertaken using Stata/MP 16.0 (StataCorp LLC, Texas, USA).

## Results

### Soft launch

The results from the first 99 adolescent and 101 adult participants were analysed initially following the soft launch. The marginal frequencies for the BWS survey were as anticipated (Additional file 1: Table S2), and hence the soft launch sample was incorporated within the main sample.

Nonetheless, issues were observed with the adult DCE_TTO_ survey soft launch results in that the modelled utility decrements for the levels of each dimension (Additional file 1: Table S3) were somewhat greater than had been expected. The sociodemographic and health characteristics, and self-reported understanding of the soft launch adult sample did not display any discrepancies that may have contributed to this (Additional file 1: Tables S4 and S5). The researchers considered that the relatively long durations attached to each attribute (one, four, seven or ten years) may have substantially influenced participant responses to the DCE_TTO_, and participants may have felt these durations were somewhat implausible. Following involvement of adult PPI representatives for the wider study, the DCE_TTO_ survey was adjusted so that the duration attribute for the tasks contained shorter time periods (6 months, one year, one year and six months, two years).

As the DCE_TTO_ survey had been adjusted, the data obtained during the soft launch were excluded from further analyses.

### Main survey

A total of 723 adolescents completed the BWS survey (including the soft launch sample) and 626 adults completed the DCE_TTO_ survey.

The time taken for adolescents to complete the BWS survey ranged from 2 to 272 min, with a median time of 8 min. The time taken for adults to complete the DCE_TTO_ survey ranged between 2 and 95 min, with a median time of 8 min. It was not possible to determine the proportion of time that participants spent on completing the valuation tasks.

### Sociodemographic and health characteristics

Sociodemographic and health characteristics are provided in Table 2, alongside population norms derived from the 2011 UK Census for reference [42]. The samples were broadly representative of the sociodemographic characteristics of the UK population. The samples comprised participants from each of the devolved nations, though a smaller proportion of participants from Scotland than population norms suggest. It was not possible to locate a small proportion of participants, as 8.3% (n = 60) did not know their postcode, and 2.6% (n = 19) provided a postcode that was not recognised.

**Table 2:** Sociodemographic characteristics of the sample and population norms

Sociodemographic characteristics	Adolescents n = 723 (%)	Adults n = 626 (%)	Population norms%
**Gender**			
Male	387 (53.5)	288 (46.0)	49.1^a^
Female	333 (46.1)	336 (53.7)	50.9^a^
Other	3 (0.4)	2 (0.3)	–
**Country of residence**			
England	588 (78.7)	519 (82.9)	84.3^a^
Scotland	34 (4.7)	36 (5.8)	8.2^a^
Wales	28 (3.9)	33 (5.3)	4.7^a^
Northern Ireland	13 (1.8)	10 (1.6)	2.8^a^
Unknown	79 (10.9)	28 (4.5)	–
**Age**			
11	106 (14.7)	–	15.9^b^
12	124 (17.2)	–	16.3^b^
13	152 (21.0)	–	16.6^b^
14	126 (17.4)	–	16.9^b^
15	123 (17.0)	–	17.1^b^
16	90 (12.5)	–	17.1^b^
18–24	–	72 (11.5)	12.0^c^
25–34	–	124 (19.8)	17.0^c^
35–44	–	112 (17.9)	17.7^c^
45–64	–	191 (30.5)	32.5^c^
65+	–	127 (20.3)	20.8^c^
Prefer not to say	2 (0.3)	–	
**Ethnicity**			
White	609 (84.2)	563 (89.9)	87.2^a^
Asian/Asian British	62 (8.6)	42 (6.7)	6.2^a^
Black/African/Caribbean/Black British	20 (2.8)	9 (1.4)	3.0^a^
Mixed/Multiple ethnic groups	27 (3.7)	7 (1.1)	–
Other ethnic group	4 (0.6)	4 (0.6)	2.9^a^
Prefer not to say	1 (0.1)	1 (0.2)	–
**Main activity**			
In employment or self-employment	–	343 (54.8)	61.7^d^
Retired	–	133 (21.3)	13.9^d^
Housework	–	43 (6.9)	4.3^d^
Student	–	47 (7.5)	9.3^d^
Seeking work/unemployed	–	30 (4.8)	4.4^d^
Long term sick	–	25 (4.0)	4.3^d^
Prefer not to say	–	1 (0.2)	–
Other	–	4 (0.6)	2.2^d^
**Marital status**			
Single	–	178 (28.4)	35.9^a^
Married/partner	–	363 (58.0)	47.0^a^
Separated/divorced	–	57 (9.1)	7.7^a^
Widowed	–	25 (4.0)	9.4^a^
Prefer not to say	–	3 (0.5)	–
**Deprivation deciles (IMD)**			
1 (most deprived)	80 (11.1)	51 (8.2)	–
2	69 (9.5)	61 (9.7)	–
3	72 (10.0)	63 (10.1)	–
4	81 (11.2)	73 (11.7)	–
5	51 (7.1)	61 (9.7)	–
6	62 (8.6)	54 (8.6)	–
7	65 (9.0)	56 (9.0)	–
8	54 (7.5)	67 (10.7)	–
9	58 (8.0)	57 (9.1)	–
10 (least deprived)	52 (7.2)	55 (8.8)	–
Postcode not provided	60 (8.3)	17 (2.7)	–
Postcode not recognised	19 (2.6)	11 (1.8)	–

^a^Proportion of total UK population

^b^Proportion of UK adolescents aged 11–16

^c^Proportion of UK adult population (aged over 18 years)

^d^Proportion of English adult population (aged over 16 years)

A variety of ethnicities were represented within the samples, though the adult sample was less diverse than the adolescent sample, with over 90% (n = 659) of participants describing themselves as White. Nonetheless, this did not differ from the Census data substantially (87.2% White) and hence was not considered to be significant. Population data were unavailable for mixed or multiple ethnic groups as this classification of ethnicity did not align with the Census data, though it is possible that ethnic groups were underrepresented. Whilst there was a range in the levels of deprivation within the sample, as determined by the most recent Indices of Multiple Deprivation for each devolved nation, almost half (48.8%) resided in the most deprived five deciles of the UK [37–40]. The adult sample had a higher proportion of participants who described their main activity as retired or housework than is reflected in the wider population, though this is to be expected with surveys, and was not considered to be significant.

The self-reported general and dental health of adolescents that participated in these surveys (Additional file 1: Table S6) indicate that over half of the adolescents reported their general health to be very good (53%), whilst no adolescent participants reported their health to be very bad. Almost two thirds of adolescents reported no problems with their teeth (62%), whilst the remainder felt their teeth were ‘a bit’ or ‘a lot’ of a problem (34% and 3% respectively).

Adult participants reported poorer general health than the adolescent sample, with only 20% describing it to be very good. Similarly, 51% of the adult participants reported problems with their teeth. Approximately half of adolescents (48%), and the majority of adults (80%) reported previous experience of caries, through having a filling or a tooth taken out. CARIES-QC impacts were more commonly observed in the adult sample than the adolescent sample.

### Difficulty and understanding

The majority of participants in both samples found the tasks easy to understand (69% adolescents, 72% adults), though over a third of adults (39%) reported that they found it difficult to choose an answer, compared to just under 10% of adolescents (see Additional file 1: Table S7). This indicates that whilst most participants understood the tasks, a proportion of adult participants found it difficult to choose which DCE_TTO_ health profile they preferred. Nonetheless, most adults passed the dominance test (92%).

### Marginal frequencies for BWS

Marginal frequencies for the BWS survey (see Additional file 1: Table S8) revealed that the dimension most consistently rated as best by adolescents in this sample, was ‘my teeth do not hurt me at all’ (rated best 61.4% of the times it was presented). The dimension most consistently rated as worst by adolescents was ‘My teeth make me cry a lot’, which was rated as worst 50.2% of the times it was presented.

### Modelling BWS

The estimated regressions from the conditional logit model can be seen in Table 3. Hurt1 (‘not at all’) was chosen as the reference level for the model as it had the highest marginal frequency for ‘best’. The coefficients are all seen to be negative and significant. Each worsening level for each attribute has a lower value than the previous level, demonstrating that the model is fully consistent.

**Table 3:** Estimated regressions from the conditional logit model using data from the BWS survey for CARIES-QC-U

Variables	Standard model
Hurt1	–
	–
Hurt2	− 2.235***
	(0.000)
Hurt3	− 3.406***
	(0.000)
Annoy1	− 0.959***
	(0.000)
Annoy2	− 1.989***
	(0.000)
Annoy3	− 2.720***
	(0.000)
Awake1	− 0.866***
	(0.000)
Awake2	− 2.322***
	(0.000)
Awake3	− 2.827***
	(0.000)
Eat1	− 0.949***
	(0.000)
Eat2	− 1.874***
	(0.000)
Eat3	− 2.543***
	(0.000)
Cry1	− 0.266***
	(0.000)
Cry2	− 2.039***
	(0.000)
Cry3	− 3.097***
	(0.000)
Observations	56,870
Log likelihood	− 14,362
Rho^2^	0.215

*Hurt1* my teeth do not hurt me at all, *Hurt2* my teeth hurt me a bit, *Hurt3* my teeth hurt me a lot, *Annoy1* my teeth do not annoy me at all, *Annoy2* my teeth annoy me a bit, *Annoy3* my teeth annoy me a lot, *Awake1* my teeth do not keep me awake at all, *Awake2* my teeth keep me awake a bit, *Awake3* my teeth keep me awake a lot, *Eat1* my teeth do not make it hard to eat some foods, *Eat2* my teeth make it a bit hard to eat some foods, *Eat3* my teeth make it really hard to eat some foods, *Cry1* my teeth do not make me cry at all, *Cry2* my teeth make me cry a bit, *Cry3* my teeth make me cry a lot

*p* values are in parentheses, where ***p < 0.01, **p < 0.05, *p < 0.1

The largest decrements at the lower severity levels can be seen for Awake2 and Cry2, suggesting that the movement from having no problems in these dimensions to some problems has a larger impact for these dimensions than for the other dimensions. The largest decrements for the most severe level are observed for Cry3, closely followed by Hurt3, indicating that these have the largest impact on utility when at the most severe level and hence have the largest relative importance across the dimensions.

Heterogeneity was explored using the reduced sample approach in relation to participant age, gender, self-reported general and dental health, and previous caries experience. The direction and significance of these coefficients (Additional file 1: Table S9 and S10) were reviewed for differences. The coefficients remained similar for each model and all values remained negative and significant, with the exception of the coefficients for Cry1. These were no longer found to be significant for models including only 11-, 12-, and 14-year-old adolescents. Similarly, the Cry1 coefficients were no longer significant for models including only participants who reported themselves as having bad or very bad general health, current dental problems or previous experience of dental caries. There were a number of anomalies present in the model estimated for participants with bad or very bad health, though this may have been impacted by the small sample of participants with this health characteristic.

### Modelling DCE_TTO_

The results from the conditional logit model can be seen in Table 4. All attribute coefficients had the expected sign (negative) except Annoy2, which was positive but insignificant (*p* = 0.820). All other coefficients were significant (*p* =  ≤ 0.05). A fully consistent model was estimated, merging levels 1 and 2 due to the importance of ensuring the utility decrement is larger for level 3 coefficients compared to level 2 coefficients within their respective dimension. This approach was supported by Chi squared tests that indicated that Annoy levels 2 and 3 were significantly different (χ^2^ = 58.23; *p* = 0.000), as were all other level 2 and 3 coefficients within each dimension.

**Table 4:** Regression results and anchored utility decrements for the standard model and the fully consistent model using data from the DCE_TTO_ survey for CARIES-QC-U

Variables	Model coefficients		Variables	Anchored values	
	Standard model	Fully consistent model		Standard model	Fully consistent model
Hurt2_LY	− 0.373***	− 0.374***	Hurt2	− 0.173	− 0.173
	(0.000)	(0.000)			
Hurt3_LY	− 1.217***	− 1.217***	Hurt3	− 0.564	− 0.562
	(0.000)	(0.000)			
Annoy2_LY	0.009	–	Annoy2	0.004	
	(0.820)	−			
Annoy3_LY	− 0.262***	− 0.266***	Annoy3	− 0.121	− 0.123
	(0.000)	(0.000)			
Awake2_LY	− 0.209***	− 0.210***	Awake2	− 0.097	− 0.097
	(0.000)	(0.000)			
Awake3_LY	− 0.634***	− 0.634***	Awake3	− 0.293	− 0.293
	(0.000)	(0.000)			
Eat2_LY	− 0.126***	− 0.126***	Eat2	− 0.058	− 0.058
	(0.000)	(0.000)			
Eat3_LY	− 0.354***	− 0.355***	Eat3	− 0.164	− 0.164
	(0.000)	(0.000)			
Cry2_LY	− 0.215***	− 0.215***	Cry2	− 0.099	− 0.099
	(0.000)	(0.000)			
Cry3_LY	− 0.565***	− 0.565***	Cry3	− 0.262	− 0.261
	(0.000)	(0.000)			
LY	2.160***	2.166***	–	–	–
	(0.000)	(0.000)			
Observations	13,086	13,086			
Log likelihood	− 3468	− 3468			
Rho^2^	0.235	0.235			

*p* values are in parentheses, where ***p < 0.01, **p < 0.05, *p < 0.1

An underscore (_) represents an interaction between variables i.e. Hurt2_LY is Hurt2 multiplied by LY. Hurt2: my teeth hurt me a bit; Hurt3: my teeth hurt me a lot; Annoy2: my teeth annoy me a bit; Annoy3: my teeth annoy me a lot; Awake2: my teeth keep me awake a bit; Awake3: my teeth keep me awake a lot; Eat2: my teeth make it a bit hard to eat some foods; Eat3: my teeth make it really hard to eat some foods; Cry2: my teeth make me cry a bit; Cry3: my teeth make me cry a lot; LY: duration

On reviewing the anchored coefficients for the fully consistent model, the largest utility decrement can be seen for Hurt3, suggesting this item has the greatest relative importance. Conversely, Eat3 had the smallest utility decrement, suggesting this item has the least relative importance. Amongst the level 2 coefficients Hurt2 also has the largest utility decrement, demonstrating the large relative impact on utility from the Hurt dimension.

The assumption that the duration attribute was linear was confirmed through a test of linearity where the duration variables were entered into the regression as dummy variables. When plotted, the life years coefficients for the dummy variables form a straight line (Additional file 1: Figure S1).

The inclusion of interaction terms were explored to determine whether gender, age employment status, marital status, general health, the presence of existing dental problems and previous caries experience had an impact on the preferences provided. Positive attributes were identified for almost all dimensions for participants with self-reported current dental problems, demonstrating that these participants gave higher values than those without current dental problems. Conversely, a number of negative interactions were seen for participants over the age of 65 years, suggesting older participants gave lower values for these dimension levels. The interaction effects can be viewed in Additional file 1: Table S11.

#### Model robustness

Regressions were estimated for seven further models based upon the BWS and DCE_TTO_ models respectively, each with a reduced sample. These excluded participants that failed the dominance test, reported difficulty understanding the tasks or difficulty in choosing an answer within the tasks, and combinations of these. These also explored robustness when excluding those that completed the survey in less than 3 min, and more than 30 min. The regressions can be found in Additional file 1: Tables S12–S16. The models were seen to produce minimal changes in the regressions estimated, suggesting the standard baseline models were robust, with the exception of Annoy2 in which the coefficient changed from being positive to negative in the DCE_TTO_ regressions. Whilst this beneficial change was observed in most of the additional DCE_TTO_ models (robustness models 1–5), it was not significant.

### Anchoring adolescent BWS values onto the QALY scale

The estimated regressions mapping the BWS values onto DCE_TTO_ values, and the models exploring the inclusion of squared (quadratic) and cubic terms, can be seen in Table 5. Mean absolute error between the observed and predicted values indicates that a large proportion of the predictions were greater than 0.05 or 0.1 from the observed values. The quadratic model has the fewest predictions with error greater than 0.05 or 0.1 (note that predictions were capped at 1 since utilities cannot be greater than 1).

**Table 5:** Mapping models used to generate health state utility values using adolescent BWS preferences

Variables	Anchored DCE_TTO_ utilities		
	Linear	Squared	Cubed
Modelled BWS value linear	0.119***	1.047*	0.079
	(0.000)	(0.070)	(0.471)
Modelled BWS value squared	–	-0.004***	-0.000
	–	(0.006)	(0.992)
Modelled BWS value cubed	–	–	0.000
	–	–	(0.763)
Constant	1.503***	1.194***	1.280***
	(0.000)	(0.000)	(0.000)
Mean absolute difference	0.113	0.110	0.110
Number of predictions > 0.05 from observed DCE_TTO_	181	177	180
Number of predictions > 0.1 from observed DCE_TTO_	124	114	115
Observations	243	243	243
R-squared	0.788	0.795	0.795

*p* values are in parentheses, where ***p < 0.01, **p < 0.05, *p < 0.1

Plots of the observed and predicted utilities (Additional file 1: Figures S2–S4) also show that the quadratic model generates utility values that most closely follow the pattern of modelled DCE_TTO_ values. For this reason, the quadratic model is selected as the recommended model. The adolescent and adults value sets shown in Additional file 1: Table S17 can be used directly to score CARIES-QC-U health states in cost-utility analyses.

## Discussion

This paper describes the valuation of CARIES-QC-U; a child-centred caries-specific PBM. This study has obtained preferences from adolescents using BWS and mapped these values onto the QALY scale using adult values obtained from a DCE_TTO_ survey. This has allowed the generation of utility values for all health states defined by the CARIES-QC-U classification system. The involvement of children and young people as PPI representatives, members of the steering group and active participants has been integral to the development of this PBM. CARIES-QC-U can now be used to estimate utility values in order to calculate QALYs for assessing the cost-effectiveness of new and existing interventions to prevent and manage dental caries in children. Whilst the emphasis of this paper is the generation of an adolescent value set, it is important to note that this study has also generated an adult value set which was valued by a representative sample of the UK general population, as recommended to inform decision-making for agencies such as the National Institute for Health and Care Excellence (NICE). The decision as to which value set to use is normative.

The results suggest that adolescents felt *‘hurt’* to be the most important attribute in CARIES-QC-U. The majority of participants tended to place more weight on the attribute relating to their teeth hurting a lot (Hurt3), as this dimension level was found to have the largest utility decrement in this sample. Conversely, participants valued the no impairment level of this attribute (Hurt1) most highly as indicated by the marginal frequencies.

The second largest utility decrement related to participants crying about their teeth a lot (Cry3). The importance of this attribute, is not surprising, particularly in the context of an adolescent population. The adolescent valuation of CHU9D found that adolescents placed far greater importance on what the authors termed ‘mental health attributes’ (a dimension comprising the attributes *‘worried’*, *‘sad’* and *‘annoyed’*) than adults [13, 44, 49]. In line with the CHU9D findings, the present study found that adults placed greater emphasis on the physical impacts of caries, primarily the *‘hurt’* dimension.

Previous BWS studies have used approaches to scale the coefficients, to allow the PITS state (in this case, the state with the lowest OHRQoL specific to caries: 33333) to represent 0 and the state with no impacts (11111) to be placed at 1 [41]. Whilst this approach was explored, it was not considered necessary for the present study due to the use of mapping techniques instead to anchor at dead (0).

The utility decrement from the adult survey for a lot of dental pain (Hurt3) was larger than anticipated. A disutility of 0.56 is similar to what other studies have reported for health states in considerably more severe, systemic and life-threatening conditions such as cancer [50, 51]. This may be due to the duration levels used in the DCE_TTO_ survey, since the DCE_TTO_ survey involved profiles with toothache, unchanging, for 2 years. Toothache is recognised to be a debilitating pain, though measurement of dental pain is open to considerable subjectivity [52]. Approximately 80% of the adult sample reported receipt of treatment for dental caries; either a filling or an extraction, and almost half of the adult participants reported some degree of current problems relating to their dentition. As such, it is highly likely that a substantial proportion of adult participants had previously experienced toothache, or were experiencing it at the time they completed the survey. For these participants, the thought of experiencing the same severity of pain for the durations stated in the tasks was understandably likely to be considered extremely unpleasant. This may provide an explanation for the notably high utility decrement related to pain for adult participants, as described below.

The use of a full factorial experimental design for the BWS study, efforts to improve accessibility of the surveys, and the adoption of an inclusive approach to avoid exclusion of any participants on the basis of engagement and understanding can be considered strengths of this study. Nonetheless, this study had a number of limitations.

As the samples for these surveys were identified from a survey platform, the participants are likely to regularly complete surveys such as this and may have developed skills and expertise in this process. Despite efforts to identify nationally representative samples, a degree of selection bias will exist, whereby these participants may not reflect the views of the wider population who do not regularly engage with surveys of this type, or those who do not own a computer. Furthermore, it is possible that individuals with dental problems may have self-selected into the study due to their personal interest in the topic.

Whilst other studies have recommended that the use of disease labels in health state valuation surveys is avoided [53–55], this was not possible in the present study due to the nature of the CARIES-QC instrument and the involvement of a younger population. Nonetheless, the authors acknowledge that this approach can allow respondents to bring their own experiences and potentially pre-existing misconceptions to the task [55].

The online nature of the surveys meant that it was not possible to confirm whether the BWS survey was indeed completed by the adolescent and not by their parent. Similarly, the extent of parental influence over adolescents as they completed the survey could not be determined.

Finally, the use of adult values to anchor the preferences of adolescents is not considered ideal, particularly in a child-centred study, though unfortunately there was no other feasible option.

## Conclusion

This paper makes a valuable contribution to the literature, presenting the valuation of the first utility measure specifically designed for application in dentistry, moreover the first designed specifically for a paediatric population. The adolescent and adult value sets produced are able to provide a utility for every health state defined by the CARIES-QC-U classification system. After validation, there are a wealth of potential applications for the use of CARIES-QC-U in determining the cost-effectiveness of interventions to improve children’s oral health. The measure will be of particular use in economic evaluations to determine the most cost-effective pathways for managing children with caries across primary and secondary care, with the ultimate goal of reducing the number of general anaesthetics required for treating this condition, whilst improving the quality and timing of those that are required.

## Supplementary Information

**Additional file 1**. Supplemental materials.

## Data Availability

The authors believe all relevant data have been provided in the manuscript and comprehensive Additional file 1, though any additional data are available on request from the authors.

## Abbreviations

BWS: Best–worst scaling
CARIES-QC: Caries Impacts and Experiences Questionnaire for Children
CARIES-QC-U: Caries Impacts and Experiences Questionnaire for Children Utility version
CHU9D: Child health utility 9 dimensions
DCE: Discrete choice experiment
DCE_TTO_: Discrete choice experiment/time trade-off hybrid
EQ-5D-Y: Euroqol 5 dimensions youth version
NICE: The National Institute for Health and Care Excellence
OHRQoL: Oral health-related quality of life
PBM: Preference-based measure
QALY: Quality adjusted life year
SG: Standard gamble
TTO: Time trade-off
