# Supplementary material for: Adolescent valuation of CARIES-QC-U: a child-centred preference-based measure of dental caries
Source: Health Qual Life Outcomes. 2022 Feb 3;20:18. doi: 10.1186/s12955-022-01918-w (PMC8812216; doi:10.1186/s12955-022-01918-w)
Supplement: Supplementary file 1 — Additional file 1. Supplemental materials. [file 12955_2022_1918_MOESM1_ESM.pdf]

Valuation of CARIES-QC-U: a child-centred preference-based measure of dental caries

Rogers HJ,[1] Sagebiel J,[2] Marshman Z,[3] Rodd HD,[3] Rowen D[4]

1) School of Dental Sciences, Faculty of Medical Sciences, Newcastle University, Newcastle-upon-Tyne, UK
2) Julian Sagebiel, Department of Economics, Swedish University of Agricultural Economics, Uppsala, Sweden
3) Unit of Oral Health, Dentistry and Society, School of Clinical Dentistry, University of Sheffield, UK
4) Health Economics and Decision Science, School of Health and Related Research, University of Sheffield, UK

Corresponding author: Helen J Rogers (ORCID: 0000-0001-5031-5009), School of Dental Sciences, Newcastle University, UK. Email: helen.rogers@newcastle.ac.uk

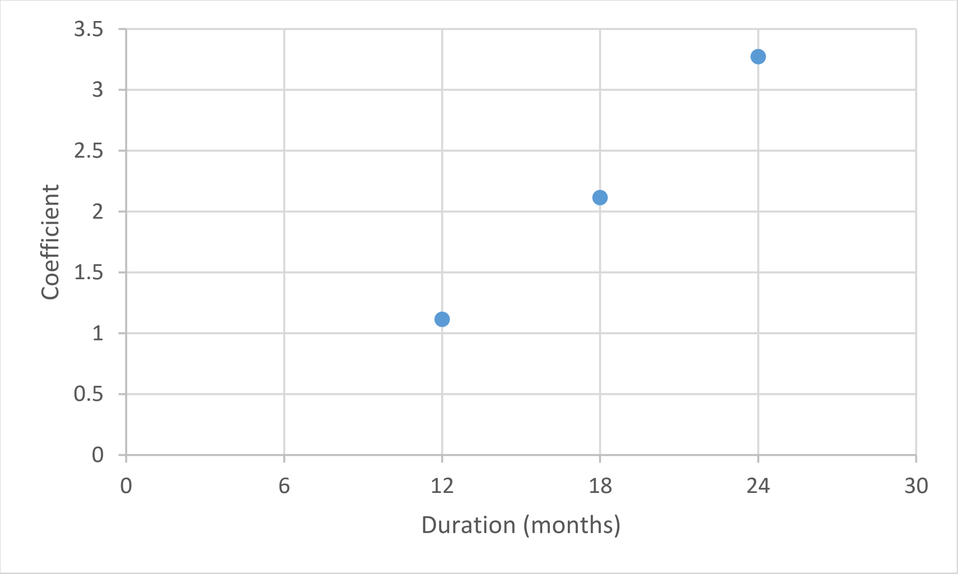

Figure S1: Plot demonstrating linearity of the duration attribute in the DCE_{TTO} tasks

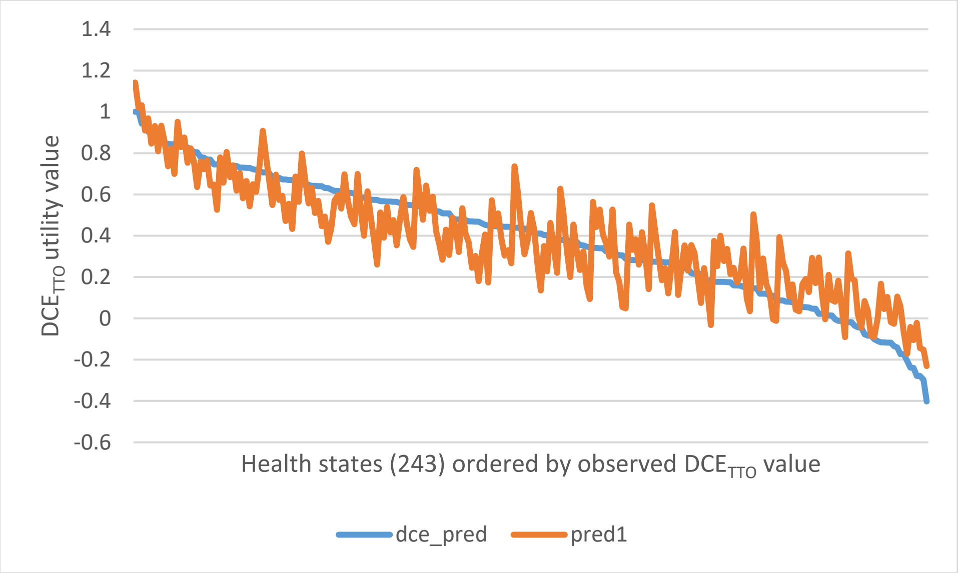

Figure S2: Plot showing the relationship between the estimated and observed values for the linear model

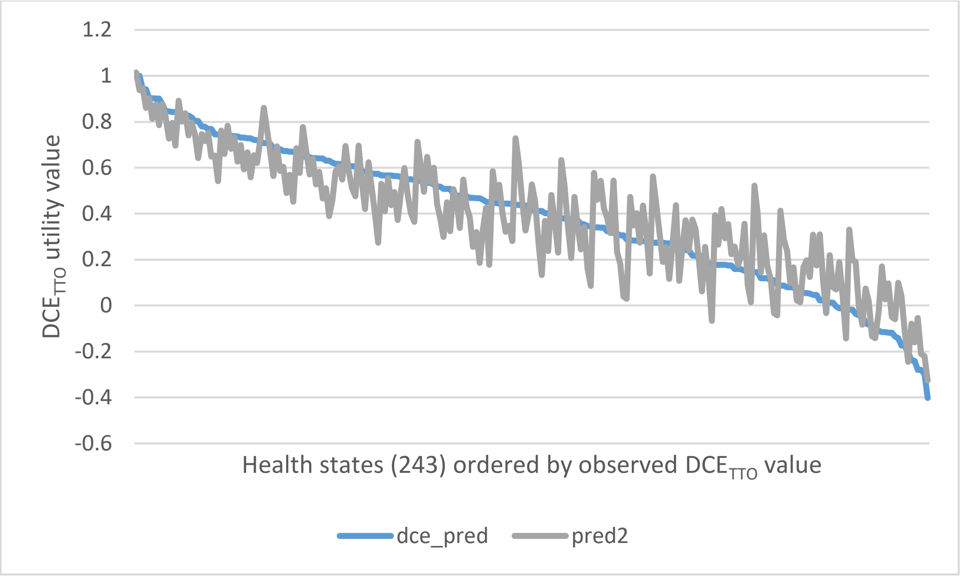

Figure S3: Plot showing the relationship between the estimated and observed values using the quadratic model

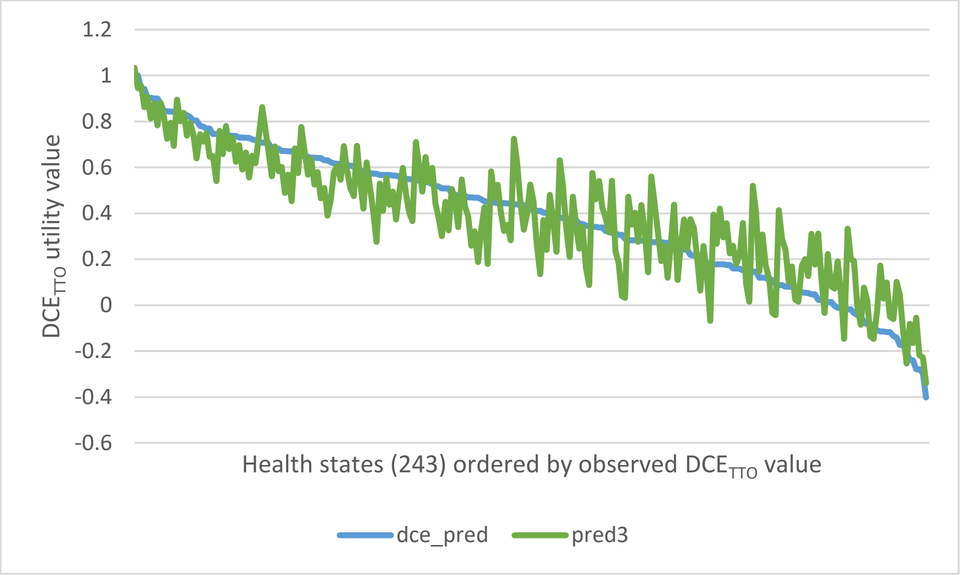

Figure S4: Plot showing the relationship between the estimated and observed values using the cubic model

Table S1: CARIES-QC contains the 13 questions shown here alongside the related items and response levels (reproduced from Rogers et al, 2020)

Question from CARIES-QC	Item	Response levels
How much do your teeth hurt you?	Hurt	Not at all<br>A bit<br>A lot
Do your teeth make it hard to eat some foods?	Hard to eat	Not at all<br>A bit<br>A lot
Do you have to eat on one side of your mouth because of your teeth?	One side	Not at all<br>A bit<br>A lot
Do you get food stuck in your teeth?	Food stuck	Not at all<br>A bit<br>A lot
How much do you get kept awake by your teeth?	Kept awake	Not at all<br>A bit<br>A lot
How much do your teeth annoy you?	Annoy	Not at all<br>A bit<br>A lot
How much do your teeth hurt when you brush them?	Brushing	Not at all<br>A bit<br>A lot
Do you have to eat more carefully because of your teeth?	Carefully	Not at all<br>A bit<br>A lot
Do you have to eat more slowly because of your teeth?	Slowly	Not at all<br>A bit<br>A lot
Do you feel cross because of your teeth?	Cross	Not at all<br>A bit<br>A lot
How much have you cried because of your teeth?	Cried	Not at all<br>A bit<br>A lot
Do your teeth make it hard to do your schoolwork?	School	Not at all<br>A bit<br>A lot
How much of a problem are your teeth for you?	Global	Not at all<br>A bit<br>A lot

Table S2: Marginal choice frequencies for data obtained from soft launch of adolescent BWS survey

Attribute	Descriptor	Best	Worst
**Hurt1**	My teeth do not hurt me at all	**0.602**	0.050
**Hurt2**	My teeth hurt me a bit	0.068	0.166
**Hurt3**	My teeth hurt me a lot	0.030	0.474
**Annoy1**	My teeth do not annoy me at all	0.479	0.114
**Annoy2**	My teeth annoy me a bit	0.183	0.087
**Annoy3**	My teeth annoy me a lot	0.090	0.195
**Awake1**	My teeth do not keep me awake at all	0.328	0.073
**Awake2**	My teeth keep me awake a bit	0.048	0.160
**Awake3**	My teeth keep me awake a lot	0.050	0.307
**Eat1**	My teeth do not make it hard to eat some foods	0.350	0.120
**Eat2**	My teeth make it a bit hard to eat some foods	0.171	0.159
**Eat3**	My teeth make it really hard to eat some foods	0.093	0.272
**Cry1**	My teeth do not make me cry at all	0.466	0.071
**Cry2**	My teeth make me cry a bit	0.027	0.224
**Cry3**	My teeth make me cry a lot	0.027	**0.521**

Notes: Highest best and worst frequencies are displayed in **bold.** Hurt1: my teeth do not hurt me at all; Hurt2:my teeth hurt me a bit; Hurt3: my teeth hurt me a lot; Annoy1: my teeth do not annoy me at all; Annoy2: my teeth annoy me a bit; Annoy3: my teeth annoy me a lot; Awake1: my teeth do not keep me awake at all; Awake2: my teeth keep me awake a bit; Awake3: my teeth keep me awake a lot; Eat1: my teeth do not make it hard to eat some foods; Eat2: my teeth make it a bit hard to eat some foods; Eat3: my teeth make it really hard to eat some foods; Cry1: my teeth do not make me cry at all; Cry2: my teeth make me cry a bit; Cry3: my teeth make me cry a lot

Table S3: Regression results and anchored utility decrements using soft launch data of DCE_{TTO} survey for CARIES-QC-U

Variables	Estimated regressions	Anchored values
**Hurt2_LY**	-0.066***	-0.172
	(0.000)	
**Hurt3_LY**	-0.228***	-0.590
	(0.000)	
**Annoy2_LY**	0.001	0.002
	(0.978)	
**Annoy3_LY**	-0.049**	-0.126
	(0.016)	
**Awake2_LY**	-0.020	-0.051
	(0.314)	
**Awake3_LY**	-0.114***	-0.295
	(0.000)	
**Eat2_LY**	-0.026	-0.068
	(0.207)	
**Eat3_LY**	-0.072***	-0.188
	(0.001)	
**Cry2_LY**	-0.050**	-0.130
	(0.014)	
**Cry3_LY**	-0.146***	-0.378
	(0.000)	
**LY**	0.386***	
	(0.000)	
**Observations**	1,818	
**Log likelihood**	-500.6	
**Rho-squared**	0.205	

Notes: p-values are in parentheses, where *** p<0.01, ** p<0.05, * p<0.1. An underscore (_) represents an interaction between variables i.e. Hurt2_LY is Hurt2 multiplied by LY. Hurt2:my teeth hurt me a bit; Hurt3: my teeth hurt me a lot; Annoy2: my teeth annoy me a bit; Annoy3: my teeth annoy me a lot; Awake2: my teeth keep me awake a bit; Awake3: my teeth keep me awake a lot; Eat2: my teeth make it a bit hard to eat some foods; Eat3: my teeth make it really hard to eat some foods; Cry2: my teeth make me cry a bit; Cry3: my teeth make me cry a lot; LY: duration

Table S4: Sociodemographic characteristics of adult participants in the soft launch of the DCE_{TTO} survey

Sociodemographic and health characteristics	Adults n=99 (%)	Population norms %
**Gender**		
Male	58 (57.4)	49.1[a]
Female	43 (42.6)	50.9[a]
Other	0 (0.00)	-
**Age**		
18-24	1 (1.0)	12.0[b]
25-34	6 (6.0)	17.0[b]
35-44	14 (13.9)	17.7[b]
45-64	50 (49.5)	32.5[b]
65+	30 (29.7)	20.8[b]
Prefer not to say	-	
**Ethnicity**		
White	96 (95.1)	87.2[a]
Asian/Asian British	0 (0.0)	6.2[a]
Black/African/Caribbean/Black British	2 (2.0)	3.0[a]
Mixed/Multiple ethnic groups	2 (2.0)	-
Other ethnic group	0 (0.0)	2.9[a]
Prefer not to say	1 (1.0)	-
**Main activity**		
In employment or self-employment	45 (44.6)	61.7[c]
Retired	32 (31.7)	13.9[c]
Housework	7 (6.9)	4.3[c]
Student	2 (2.0)	9.3[c]
Seeking work/unemployed	6 (5.9)	4.4[c]
Long term sick	5 (5.0)	4.3[c]
Prefer not to say	1 (1.0)	-
Other	3 (3.0)	2.2[c]
**Marital status**		
Single	21 (20.8)	35.9[a]
Married/partner	68 (67.3)	47.0[a]
Separated/divorced	8 (7.9)	7.7[a]
Widowed	3 (3.0)	9.4[a]
Prefer not to say	1 (1.0)	-

Notes: [a]=proportion of total UK population [b]=proportion of UK adult population (aged over 18 years) [c]=proportion of English adult population (aged over 16 years)

Table S5: Health-related characteristics of adult participants in the soft launch of the DCE_{TTO} survey

Health characteristics	Adults n=99 (%)
**Self-reported general health: in general, how would you rate your health today?**	
Very good	12 (11.9)
Good	53 (52.5)
OK	29 (28.7)
Bad	6 (5.9)
Very bad	1 (1.0)
**Self-reported dental health: how much of a problem are your teeth for you today?**	
Not at all	53 (52.5)
A bit	37 (36.6)
A lot	11 (10.9)
**Self-reported caries experience: have you ever had a filling or a tooth taken out because it had a hole or cavity?**	
Yes	85 (84.2)
No	16 (15.8)
**Participant understanding**	
**Did you find the tasks:**	
Easy to understand	68 (67.3)
Somewhere in the middle	2 (2.0)
Difficult to understand	31 (30.7)
**Did you find it:**	
Easy to choose an answer	43 (42.6)
Somewhere in the middle	18 (17.8)
Difficult to choose an answer	40 (39.6)

Table S6: Self-reported general health and dental health of participants in the adolescent BWS and adult DCE_{TTO} surveys

Health-related characteristics	Adolescents n=723 (%)	Adults n=626 (%)
**Self-reported general health: in general, how would you rate your health today?**		
Very good	383 (53.0)	126 (20.1)
Good	272 (37.6)	297 (47.4)
OK	59 (8.2)	154 (24.6)
Bad	9 (1.2)	41 (6.6)
Very bad	0 (0.0)	8 (1.3)
**Self-reported dental health: how much of a problem are your teeth for you today?**		
Not at all	450 (62.2)	316 (50.5)
A bit	249 (34.4)	271 (43.3)
A lot	24 (3.3)	39 (6.2)
**Self-reported caries experience: have you ever had a filling or a tooth taken out because it had a hole or cavity?**		
Yes	350 (48.4)	498 (79.6)
No	373 (51.6)	128 (20.5)
**How much do your teeth hurt you?**		
Not at all	573 (79.3)	452 (72.2)
A bit	134 (18.5)	163 (26.0)
A lot	16 (2.2)	11 (1.8)
**How much do your teeth annoy you?**		
Not at all	479 (66.3)	340 (54.3)
A bit	220 (30.4)	239 (38.2)
A lot	24 (3.3)	47 (7.5)
**How much do you get kept awake by your teeth?**		
Not at all	617 (85.3)	562 (89.8)
A bit	90 (12.5)	56 (9.0)
A lot	16 (2.2)	8 (1.3)
**Do your teeth make it hard to eat some foods**		
Not at all	550 (76.1)	374 (59.7)
A bit	151 (20.9)	214 (34.2)
A lot	22 (3.0)	38 (6.1)
**How much have you cried about your teeth?**		
Not at all	604 (83.5)	546 (87.2)
A bit	106 (14.7)	67 (10.7)
A lot	13 (1.8)	13 (2.1)

Table S7: Participants' self-reported difficulty of understanding, difficulty of choice and ability to pass the dominance test

Participant understanding	Adolescents n=723 (%)	Adults n=626 (%)
**Did you find the tasks:**		
Easy to understand	501 (69.3)	451 (72.0)
Somewhere in the middle	171 (23.7)	39 (6.2)
Difficult to understand	51 (7.1)	136 (21.9)
**Did you find it:**		
Easy to choose an answer	440 (60.9)	277 (44.3)
Somewhere in the middle	215 (29.7)	104 (16.6)
Difficult to choose an answer	68 (9.4)	245 (39.1)
**Dominance test**		
Pass*	389 (53.8)	578 (92.3)
Correctly identified ‘best’ attribute	604 (83.5)	-
**Second attempt requested**	**n=38**	**n=26**
Pass*	12 (31.6) of which 0 (0.0) had failed the first attempt	23 (92.0) of which 18 (78.3) had failed the first attempt
Correctly identified ‘best’ attribute	34 (89.5)	-

Notes: *both ‘best’ and ‘worst’ attributes needed to be identified correctly for adolescents to ‘pass’ the BWS dominance test. NB. There is no agreed dominance test for BWS.

Table S8: Marginal choice frequencies for data obtained from adolescent BWS survey

Variables	Descriptor	Best	Worst
**Hurt1**	My teeth do not hurt me at all	**0.614**	0.047
**Hurt2**	My teeth hurt me a bit	0.087	0.166
**Hurt3**	My teeth hurt me a lot	0.040	0.489
**Annoy1**	My teeth do not annoy me at all	0.404	0.074
**Annoy2**	My teeth annoy me a bit	0.171	0.121
**Annoy3**	My teeth annoy me a lot	0.079	0.206
**Awake1**	My teeth do not keep me awake at all	0.341	0.081
**Awake2**	My teeth keep me awake a bit	0.074	0.141
**Awake3**	My teeth keep me awake a lot	0.056	0.305
**Eat1**	My teeth do not make it hard to eat some foods	0.346	0.107
**Eat2**	My teeth make it a bit hard to eat some foods	0.156	0.168
**Eat3**	My teeth make it really hard to eat some foods	0.077	0.277
**Cry1**	My teeth do not make me cry at all	0.455	0.070
**Cry2**	My teeth make me cry a bit	0.052	0.247
**Cry3**	My teeth make me cry a lot	0.044	**0.502**

Notes: highest best and worst frequencies are displayed in **bold**. Hurt1: my teeth do not hurt me at all; Hurt2:my teeth hurt me a bit; Hurt3: my teeth hurt me a lot; Annoy1: my teeth do not annoy me at all; Annoy2: my teeth annoy me a bit; Annoy3: my teeth annoy me a lot; Awake1: my teeth do not keep me awake at all; Awake2: my teeth keep me awake a bit; Awake3: my teeth keep me awake a lot; Eat1: my teeth do not make it hard to eat some foods; Eat2: my teeth make it a bit hard to eat some foods; Eat3: my teeth make it really hard to eat some foods; Cry1: my teeth do not make me cry at all; Cry2: my teeth make me cry a bit; Cry3: my teeth make me cry a lot

Table S9: Regression results when using the reduced sample approach to explore heterogeneity in the BWS data relating to age

Variables	Standard model	11-year-olds	12-year-olds	13-year-olds	14-year-olds	15-year-olds	16-year-olds
**Hurt1**	-	-	-	-	-	-	-
	-	-	-	-	-	-	-
**Hurt2**	2.235***	2.434***	2.291***	2.084***	2.179***	2.508***	2.311***
	(0.000)	(0.000)	(0.000)	(0.000)	(0.000)	(0.000)	(0.000)
**Hurt3**	3.406***	3.418***	4.033***	2.905***	-3.476***	-3.721***	-3.656***
	(0.000)	(0.000)	(0.000)	(0.000)	(0.000)	(0.000)	(0.000)
**Annoy1**	-0.959***	-1.056***	-1.079***	-0.815***	-0.745***	-1.192***	-1.262***
	(0.000)	(0.000)	(0.000)	(0.000)	(0.000)	(0.000)	(0.000)
**Annoy2**	-1.989***	-2.107***	-2.030***	-1.627***	-1.963***	-2.335***	-2.336***
	(0.000)	(0.000)	(0.000)	(0.000)	(0.000)	(0.000)	(0.000)
**Annoy3**	-2.720***	-2.730***	-2.720***	-2.216***	-3.139***	-3.077***	-3.365***
	(0.000)	(0.000)	(0.000)	(0.000)	(0.000)	(0.000)	(0.000)
**Awake1**	-0.866***	-0.845***	-0.949***	-0.612***	-0.697***	-1.346***	-1.060***
	(0.000)	(0.000)	(0.000)	(0.000)	(0.000)	(0.000)	(0.000)
**Awake2**	-2.322***	-2.521***	-2.566***	-1.977***	-2.099***	-2.654***	-2.759***
	(0.000)	(0.000)	(0.000)	(0.000)	(0.000)	(0.000)	(0.000)
**Awake3**	-2.827***	-3.592***	-2.940***	-2.154***	-3.000***	-2.766***	-4.283***
	(0.000)	(0.000)	(0.000)	(0.000)	(0.000)	(0.000)	(0.000)
**Eat1**	-0.949***	-1.171***	-0.787***	-0.742***	-0.896***	-1.248***	-1.261***
	(0.000)	(0.000)	(0.000)	(0.000)	(0.000)	(0.000)	(0.000)
**Eat2**	-1.874***	-1.945***	-2.012***	-1.565***	-1.774***	-2.206***	-2.203***
	(0.000)	(0.000)	(0.000)	(0.000)	(0.000)	(0.000)	(0.000)
**Eat3**	-2.543***	-2.642***	-2.828***	-2.148***	-2.371***	-2.846***	-3.336***
	(0.000)	(0.000)	(0.000)	(0.000)	(0.000)	(0.000)	(0.000)
**Cry1**	-0.266***	-0.226	-0.128	-0.350***	-0.064	-0.547***	-0.421***
	(0.000)	(0.106)	(0.338)	(0.001)	(0.602)	(0.000)	(0.005)
**Cry2**	-2.039***	-2.186***	-2.194***	-1.740***	-2.017***	-2.293***	-2.150***
	(0.000)	(0.000)	(0.000)	(0.000)	(0.000)	(0.000)	(0.000)
**Cry3**	-3.097***	-2.900***	-3.698***	-2.814***	-3.200***	-3.192***	-3.705***
	(0.000)	(0.000)	(0.000)	(0.000)	(0.000)	(0.000)	(0.000)

**Observations**	56,870	8,480	9,920	12,160	10,080	9,840	7,200
**Log likelihood**	-14362	-2104	-2381	-3343	-2493	-2471	-1735
**Rho-squared**	0.215	0.235	0.259	0.158	0.236	0.224	0.263

Notes: p-values are in parentheses, where *** p<0.01, ** p<0.05, * p<0.1. Hurt1: my teeth do not hurt me at all; Hurt2:my teeth hurt me a bit; Hurt3: my teeth hurt me a lot; Annoy1: my teeth do not annoy me at all; Annoy2: my teeth annoy me a bit; Annoy3: my teeth annoy me a lot; Awake1: my teeth do not keep me awake at all; Awake2: my teeth keep me awake a bit; Awake3: my teeth keep me awake a lot; Eat1: my teeth do not make it hard to eat some foods; Eat2: my teeth make it a bit hard to eat some foods; Eat3: my teeth make it really hard to eat some foods; Cry1: my teeth do not make me cry at all; Cry2: my teeth make me cry a bit; Cry3: my teeth make me cry a lot

Table S10: Regression results when using the reduced sample approach to explore heterogeneity in the BWS data relating to participant gender, self-reported general and dental health, and previous caries experience

Variables	Standard model	Male gender	Bad or very bad general health	Current dental problems	Previous caries experience
**Hurt1**	-	-	-	-	-
	-	-	-	-	-
**Hurt2**	-2.235***	-2.272***	-3.105***	-1.594***	-1.921***
	(0.000)	(0.000)	(0.000)	(0.000)	(0.000)
**Hurt3**	-3.406***	-3.434***	-4.914***	-2.592***	-3.199***
	(0.000)	(0.000)	(0.000)	(0.000)	(0.000)
**Annoy1**	-0.959***	-0.937***	-1.580***	-0.552***	-0.733***
	(0.000)	(0.000)	(0.002)	(0.000)	(0.000)
**Annoy2**	-1.989***	-1.958***	-3.529***	-1.063***	-1.637***
	(0.000)	(0.000)	(0.005)	(0.000)	(0.000)
**Annoy3**	-2.720***	-2.632***	-4.028**	-1.670***	-2.215***
	(0.000)	(0.000)	(0.010)	(0.000)	(0.000)
**Awake1**	-0.866***	-0.830***	-1.452**	-0.460***	-0.704***
	(0.000)	(0.000)	(0.012)	(0.000)	(0.000)
**Awake2**	-2.322***	-2.263***	-3.439***	-1.395***	-1.892***
	(0.000)	(0.000)	(0.000)	(0.000)	(0.000)
**Awake3**	-2.827***	-2.584***	-19.060***	-1.773***	-2.293***
	(0.000)	(0.000)	(0.000)	(0.000)	(0.000)
**Eat1**	-0.949***	-0.937***	-1.359*	-0.430***	-0.751***
	(0.000)	(0.000)	(0.076)	(0.000)	(0.000)
**Eat2**	-1.874***	-1.790***	-2.100**	-1.148***	-1.534***
	(0.000)	(0.000)	(0.012)	(0.000)	(0.000)
**Eat3**	-2.543***	-2.360***	-3.488***	-1.678***	-2.155***
	(0.000)	(0.000)	(0.003)	(0.000)	(0.000)
**Cry1**	-0.266***	-0.302***	-0.671	-0.075	-0.216***
	(0.000)	(0.000)	(0.159)	(0.390)	(0.006)
**Cry2**	-2.039***	-2.016***	-2.549***	-1.535***	-1.841***
	(0.000)	(0.000)	(0.001)	(0.000)	(0.000)
**Cry3**	-3.097***	-2.988***	-4.593***	-2.312***	-2.857***
	(0.000)	(0.000)	(0.000)	(0.000)	(0.000)

**Observations**	56,870	30,960	720	21,840	28,000
**Log likelihood**	-14362	-8019	-145.8	-6277	-7535
**Rho-squared**	0.215	0.201	0.371	0.120	0.171

Notes: p-values are in parentheses, where *** p<0.01, ** p<0.05, * p<0.1. Hurt1: my teeth do not hurt me at all; Hurt2:my teeth hurt me a bit; Hurt3: my teeth hurt me a lot; Annoy1: my teeth do not annoy me at all; Annoy2: my teeth annoy me a bit; Annoy3: my teeth annoy me a lot; Awake1: my teeth do not keep me awake at all; Awake2: my teeth keep me awake a bit; Awake3: my teeth keep me awake a lot; Eat1: my teeth do not make it hard to eat some foods; Eat2: my teeth make it a bit hard to eat some foods; Eat3: my teeth make it really hard to eat some foods; Cry1: my teeth do not make me cry at all; Cry2: my teeth make me cry a bit; Cry3: my teeth make me cry a lot

Table S11: The impact of sociodemographic and health-related interaction terms in the DCE_{TTO} survey results

	Male participants	Self-reported current dental problems	Previous caries experience	Employed participants	Married participants	Participants with bad or very bad general health	Participants aged 65 and over	Participants aged 18-24
**Hurt2_LY**		+			+	-		
**Hurt3_LY**	+	+			+	-	-	
**Annoy2_LY**		+						
**Annoy3_LY**		+					-	
**Awake2_LY**								
**Awake3_LY**	+	+						
**Eat2_LY**		+						
**Eat3_LY**		+		+				-
**Cry2_LY**		+					-	
**Cry3_LY**		+		+			-	

Notes: + a positive, statistically significant ($p \leq 0.05$) coefficient – a negative, statistically significant ($p \leq 0.05$) coefficient. An underscore (_) represents an interaction between variables i.e. Hurt2_LY is Hurt2 multiplied by LY. Hurt2: my teeth hurt me a bit; Hurt3: my teeth hurt me a lot; Annoy2: my teeth annoy me a bit; Annoy3: my teeth annoy me a lot; Awake2: my teeth keep me awake a bit; Awake3: my teeth keep me awake a lot; Eat2: my teeth make it a bit hard to eat some foods; Eat3: my teeth make it really hard to eat some foods; Cry2: my teeth make me cry a bit; Cry3: my teeth make me cry a lot; LY: duration

Table S12: Regression results exploring the BWS model robustness to exclusion of adolescent participants who failed the dominance test, difficulty understanding or difficulty of choice

Variables	Standard model	Robustness 1<br>Excluding participants that failed the dominance test† first time	Robustness 2<br>Excluding participants that found it difficult to understand the tasks	Robustness 3<br>Excluding participants that failed the dominance test† and found it difficult to understand the tasks	Robustness 4<br>Excluding participants that found it difficult to choose an answer	Robustness 5<br>Excluding participants that failed the dominance test† and found it difficult to choose an answer
**Hurt1**	-	-	-	-	-	-
	-	-	-	-	-	-
**Hurt2**	-2.235***	-3.322***	-2.361***	-3.383***	-2.389***	-3.411***
	(0.000)	(0.000)	(0.000)	(0.000)	(0.000)	(0.000)
**Hurt3**	-3.406***	-4.918***	-3.614***	-5.132***	-3.641***	-5.178***
	(0.000)	(0.000)	(0.000)	(0.000)	(0.000)	(0.000)
**Annoy1**	-0.959***	-1.569***	-1.018***	-1.569***	-1.036***	-1.583***
	(0.000)	(0.000)	(0.000)	(0.000)	(0.000)	(0.000)
**Annoy2**	-1.989***	-3.418***	-2.146***	-3.482***	-2.172***	-3.500***
	(0.000)	(0.000)	(0.000)	(0.000)	(0.000)	(0.000)
**Annoy3**	-2.720***	-4.619***	-2.922***	-4.643***	-2.968***	-4.696***
	(0.000)	(0.000)	(0.000)	(0.000)	(0.000)	(0.000)
**Awake1**	-0.866***	-1.517***	-0.929***	-1.513***	-0.947***	-1.532***
	(0.000)	(0.000)	(0.000)	(0.000)	(0.000)	(0.000)
**Awake2**	-2.322***	-3.938***	-2.508***	-3.992***	-2.544***	-4.018***
	(0.000)	(0.000)	(0.000)	(0.000)	(0.000)	(0.000)
**Awake3**	-2.827***	-5.519***	-3.055***	-5.606***	-3.085***	-5.683***
	(0.000)	(0.000)	(0.000)	(0.000)	(0.000)	(0.000)
**Eat1**	-0.949***	-1.729***	-1.027***	-1.757***	-1.063***	-1.802***
	(0.000)	(0.000)	(0.000)	(0.000)	(0.000)	(0.000)
**Eat2**	-1.874***	-3.167***	-2.016***	-3.255***	-2.037***	-3.276***
	(0.000)	(0.000)	(0.000)	(0.000)	(0.000)	(0.000)
**Eat3**	-2.543***	-4.713***	-2.731***	-4.762***	-2.767***	-4.785***
	(0.000)	(0.000)	(0.000)	(0.000)	(0.000)	(0.000)
**Cry1**	-0.266***	-0.577***	-0.292***	-0.573***	-0.285***	-0.576***

	(0.000)	(0.000)	(0.000)	(0.000)	(0.000)	(0.000)
**Cry2**	-2.039***	-3.019***	-2.122***	-3.045***	-2.136***	-3.065***
	(0.000)	(0.000)	(0.000)	(0.000)	(0.000)	(0.000)
**Cry3**	-3.097***	-4.965***	-3.306***	-5.046***	-3.345***	-5.114***
	(0.000)	(0.000)	(0.000)	(0.000)	(0.000)	(0.000)
**Observations**	56,870	31,120	53,760	29,920	52,400	29,520
**Log likelihood**	-14362	-5857	-13374	-5546	-12942	-5431
**Rho-squared**	0.215	0.415	0.235	0.424	0.239	0.428

Notes: Robust p-values are in parentheses, where *** p<0.01, ** p<0.05, * p<0.1 [†]Dominance test was failed if participant was unable to identify the correct 'best' and 'worst' attributes. Hurt2:my teeth hurt me a bit; Hurt3: my teeth hurt me a lot; Annoy2: my teeth annoy me a bit; Annoy3: my teeth annoy me a lot; Awake2: my teeth keep me awake a bit; Awake3: my teeth keep me awake a lot; Eat2: my teeth make it a bit hard to eat some foods; Eat3: my teeth make it really hard to eat some foods; Cry2: my teeth make me cry a bit; Cry3: my teeth make me cry a lot.

Table S13: Regression results exploring the BWS model robustness to exclusion of adolescent participants who completed the survey very quickly or very slowly

Variables	Standard model	Robustness 6<br>Excluding participants that completed the survey in < 3 mins	Robustness 7<br>Excluding participants that completed the survey in > 30 mins
**Hurt1**	-	-	-
	-	-	-
**Hurt2**	-2.235***	-2.263***	-2.234***
	(0.000)	(0.000)	(0.000)
**Hurt3**	-3.406***	-3.495***	-3.439***
	(0.000)	(0.000)	(0.000)
**Annoy1**	-0.959***	-0.976***	-0.967***
	(0.000)	(0.000)	(0.000)
**Annoy2**	-1.989***	-2.023***	-1.999***
	(0.000)	(0.000)	(0.000)
**Annoy3**	-2.720***	-2.773***	-2.785***
	(0.000)	(0.000)	(0.000)
**Awake1**	-0.866***	-0.878***	-0.865***
	(0.000)	(0.000)	(0.000)
**Awake2**	-2.322***	-2.356***	-2.357***
	(0.000)	(0.000)	(0.000)
**Awake3**	-2.827***	-2.896***	-2.868***
	(0.000)	(0.000)	(0.000)
**Eat1**	-0.949***	-0.966***	-0.962***
	(0.000)	(0.000)	(0.000)
**Eat2**	-1.874***	-1.903***	-1.904***
	(0.000)	(0.000)	(0.000)
**Eat3**	-2.543***	-2.587***	-2.578***
	(0.000)	(0.000)	(0.000)
**Cry1**	-0.266***	-0.270***	-0.289***
	(0.000)	(0.000)	(0.000)

**Cry2**	-2.039***	-2.064***	-2.064***
	(0.000)	(0.000)	(0.000)
**Cry3**	-3.097***	-3.174***	-3.121***
	(0.000)	(0.000)	(0.000)
**Observations**	56,870	56,230	54,400
**Log likelihood**	-14362	-14080	-13678
**Rho-squared**	0.215	0.222	0.219

Notes: p-values are in parentheses, where *** p<0.01, ** p<0.05, * p<0.1. Hurt1: my teeth do not hurt me at all; Hurt2:my teeth hurt me a bit; Hurt3: my teeth hurt me a lot; Annoy1: my teeth do not annoy me at all; Annoy2: my teeth annoy me a bit; Annoy3: my teeth annoy me a lot; Awake1: my teeth do not keep me awake at all; Awake2: my teeth keep me awake a bit; Awake3: my teeth keep me awake a lot; Eat1: my teeth do not make it hard to eat some foods; Eat2: my teeth make it a bit hard to eat some foods; Eat3: my teeth make it really hard to eat some foods; Cry1: my teeth do not make me cry at all; Cry2: my teeth make me cry a bit; Cry3: my teeth make me cry a lot

Table S14: Regression results exploring model robustness to exclusion of adult participants who failed the dominance test or had difficulty understanding the DCE_{TTO} tasks

Variables	Standard model	Standard model anchored	Robustness 1	Robustness 1 anchored	Robustness 2	Robustness 2 anchored	Robustness 3	Robustness 3 anchored
			Excluding participants that failed the dominance test		Excluding participants that found it difficult to understand the tasks		Excluding participants that failed the dominance test and found it difficult to understand the tasks	
**Hurt2_LY**	-0.373***	-.1728429	-0.425***	-.1742738	-0.389***	-.1729981	-0.398***	-.1733476
	(0.000)	(0.000)	(0.000)	(0.000)	(0.000)	(0.000)	(0.000)	(0.000)
**Hurt3_LY**	-1.217***	-.5635363	-1.354***	-.5548113	-1.283***	-.5708403	-1.315***	-.5722786
	(0.000)	(0.000)	(0.000)	(0.000)	(0.000)	(0.000)	(0.000)	(0.000)
**Annoy2_LY**	0.009	.004016	-0.034	-.0137991	-0.032	-.014017	-0.031	-.0135733
	(0.820)	(0.821)	(0.414)	(0.409)	(0.460)	(0.455)	(0.472)	(0.468)
**Annoy3_LY**	-0.262***	-.1212449	-0.313***	-.1280649	-0.289***	-.1287591	-0.302***	-.1312883
	(0.000)	(0.000)	(0.000)	(0.000)	(0.000)	(0.000)	(0.000)	(0.000)
**Awake2_LY**	-0.209***	-.0967463	-0.234***	-.0959131	-0.214***	-.0952338	-0.216***	-.0938763
	(0.000)	(0.000)	(0.000)	(0.000)	(0.000)	(0.000)	(0.000)	(0.000)
**Awake3_LY**	-0.634***	-.2933218	-0.723***	-.2960955	-0.682***	-.3034562	-0.694***	-.3022111
	(0.000)	(0.000)	(0.000)	(0.000)	(0.000)	(0.000)	(0.000)	(0.000)
**Eat2_LY**	-0.126***	-.0582052	-0.149***	-.0609352	-0.136***	-.060538	-0.138***	-.0599877
	(0.000)	(0.000)	(0.000)	(0.000)	(0.001)	(0.000)	(0.001)	(0.000)
**Eat3_LY**	-0.354***	-.1636369	-0.396***	-.1624236	-0.353***	-.1568854	-0.357***	-.1553178
	(0.000)	(0.000)	(0.000)	(0.000)	(0.000)	(0.000)	(0.000)	(0.000)
**Cry2_LY**	-0.215***	-.0994839	-0.235***	-.0962163	-0.209***	-.0930287	-0.220***	-.0956501
	(0.000)	(0.000)	(0.000)	(0.000)	(0.000)	(0.000)	(0.000)	(0.000)
**Cry3_LY**	-0.565***	-.2615666	-0.618***	-.2532607	-0.580***	-.2580093	-0.584***	-.2543272
	(0.000)	(0.000)	(0.000)	(0.000)	(0.000)	(0.000)	(0.000)	(0.000)
**LY**	2.160***	-	2.440***	-	2.248***		2.298***	
	(0.000)	-	(0.000)	-	(0.000)		(0.000)	

**Observations**	13,086	12,240	10,890	10,710
**Log likelihood**	-3468	-3118	-2830	-2760
**Rho-squared**	0.235	0.265	0.250	0.256

Notes: p-values are in parentheses, where *** p<0.01, ** p<0.05, * p<0.1. An underscore (_) represents an interaction between variables i.e. Hurt2_LY is Hurt2 multiplied by LY. Hurt2:my teeth hurt me a bit; Hurt3: my teeth hurt me a lot; Annoy2: my teeth annoy me a bit; Annoy3: my teeth annoy me a lot; Awake2: my teeth keep me awake a bit; Awake3: my teeth keep me awake a lot; Eat2: my teeth make it a bit hard to eat some foods; Eat3: my teeth make it really hard to eat some foods; Cry2: my teeth make me cry a bit; Cry3: my teeth make me cry a lot; LY: duration

Table S15: Unanchored and anchored regression results exploring model robustness to exclusion of adult participants that had difficulty choosing a response to the DCE_{TTO} tasks

Variables	Standard model	Standard model anchored	Robustness 4	Robustness 4 anchored	Robustness 5	Robustness 5 Anchored
			Excluding participants that found it difficult to choose a response		Excluding participants that found it difficult to choose a response and failed the dominance test	
**Hurt2_LY**	-0.373***	-.1728429	-0.390***	-.1762983	-0.393***	-.1761347
	(0.000)	(0.000)	(0.000)	(0.000)	(0.000)	(0.000)
**Hurt3_LY**	-1.217***	-.5635363	-1.275***	-.5755977	-1.278***	-.572701
	(0.000)	(0.000)	(0.000)	(0.000)	(0.000)	(0.000)
**Annoy2_LY**	0.009	.004016	-0.003	-.0012436	-0.008	-.003385
	(0.820)	(0.821)	(0.945)	(0.945)	(0.851)	(0.851)
**Annoy3_LY**	-0.262***	-.1212449	-0.285***	-.1288312	-0.291***	-.1304021
	(0.000)	(0.000)	(0.000)	(0.000)	(0.000)	(0.000)
**Awake2_LY**	-0.209***	-.0967463	-0.205***	-.092646	-0.203***	-.0908825
	(0.000)	(0.000)	(0.000)	(0.000)	(0.000)	(0.000)
**Awake3_LY**	-0.634***	-.2933218	-0.672***	-.303432	-0.674***	-.3020873
	(0.000)	(0.000)	(0.000)	(0.000)	(0.000)	(0.000)
**Eat2_LY**	-0.126***	-.0582052	-0.132***	-.0594476	-0.130***	-.0581255
	(0.000)	(0.000)	(0.000)	(0.000)	(0.001)	(0.000)
**Eat3_LY**	-0.354***	-.1636369	-0.363***	-.1638541	-0.363***	-.1627197
	(0.000)	(0.000)	(0.000)	(0.000)	(0.000)	(0.000)
**Cry2_LY**	-0.215***	-.0994839	-0.222***	-.1003128	-0.227***	-.1018175
	(0.000)	(0.000)	(0.000)	(0.000)	(0.000)	(0.000)
**Cry3_LY**	-0.565***	-.2615666	-0.598***	-.2700924	-0.599***	-.2683327
	(0.000)	(0.000)	(0.000)	(0.000)	(0.000)	(0.000)
**LY**	2.160***	-	2.214***		2.232***	
	(0.000)	-	(0.000)		(0.000)	
**Observations**	13,086		12,348		12,222	

**Log likelihood**	-3468	-3218	-3180
**Rho-squared**	0.235	0.248	0.249

Notes: p-values are in parentheses, where *** p<0.01, ** p<0.05, * p<0.1. An underscore (_) represents an interaction between variables i.e. Hurt2_LY is Hurt2 multiplied by LY. Hurt2:my teeth hurt me a bit; Hurt3: my teeth hurt me a lot; Annoy2: my teeth annoy me a bit; Annoy3: my teeth annoy me a lot; Awake2: my teeth keep me awake a bit; Awake3: my teeth keep me awake a lot; Eat2: my teeth make it a bit hard to eat some foods; Eat3: my teeth make it really hard to eat some foods; Cry2: my teeth make me cry a bit; Cry3: my teeth make me cry a lot; LY: duration

Table S16: Unanchored and anchored regression results exploring model robustness to exclusion of adult participants that completed the DCE_{TTO} survey very quickly or very slowly

Variables	Standard model	Standard model anchored	Robustness 6	Robustness 6 anchored	Robustness 7	Robustness 7 anchored
			Excluding participants that completed the survey in < 3 mins		Excluding participants that completed the survey in > 30 mins	
**Hurt2_LY**	-0.373***	-.1728429	-0.400***	-.1793527	-0.379***	-.175584
	(0.000)	(0.000)	(0.000)	(0.000)	(0.000)	(0.000)
**Hurt3_LY**	-1.217***	-.5635363	-1.266***	-.5679314	-1.228***	-.5683242
	(0.000)	(0.000)	(0.000)	(0.000)	(0.000)	(0.000)
**Annoy2_LY**	0.009	.004016	0.010	.0043796	0.010	.004768
	(0.820)	(0.821)	(0.803)	(0.803)	(0.791)	(0.792)
**Annoy3_LY**	-0.262***	-.1212449	-0.270***	-.1210805	-0.262***	-.1213109
	(0.000)	(0.000)	(0.000)	(0.000)	(0.000)	(0.000)
**Awake2_LY**	-0.209***	-.0967463	-0.210***	-.0940191	-0.205***	-.09466
	(0.000)	(0.000)	(0.000)	(0.000)	(0.000)	(0.000)
**Awake3_LY**	-0.634***	-.2933218	-0.657***	-.2946578	-0.639***	-.2957129
	(0.000)	(0.000)	(0.000)	(0.000)	(0.000)	(0.000)
**Eat2_LY**	-0.126***	-.0582052	-0.124***	-.055495	-0.130***	-.060248
	(0.000)	(0.000)	(0.001)	(0.000)	(0.000)	(0.000)
**Eat3_LY**	-0.354***	-.1636369	-0.358***	-.1603906	-0.356***	-.164553
	(0.000)	(0.000)	(0.000)	(0.000)	(0.000)	(0.000)
**Cry2_LY**	-0.215***	-.0994839	-0.217***	-.0972072	-0.216***	-.1001281
	(0.000)	(0.000)	(0.000)	(0.000)	(0.000)	(0.000)
**Cry3_LY**	-0.565***	-.2615666	-0.590***	-.2645564	-0.566***	-.2621079
	(0.000)	(0.000)	(0.000)	(0.000)	(0.000)	(0.000)
	2.160***	-	2.229***	-	2.161***	-
**LY**	(0.000)	-	(0.000)	-	(0.000)	-
**Observations**	13,086		12,798		12,618	
**Log likelihood**	-3468		-3346		-3342	
**Rho-squared**	0.235		0.246		0.236	

Notes: p-values are in parentheses, where *** p<0.01, ** p<0.05, * p<0.1. An underscore (_) represents an interaction between variables i.e. Hurt2_LY is Hurt2 multiplied by LY. Hurt2:my teeth hurt me a bit; Hurt3: my teeth hurt me a lot; Annoy2: my teeth annoy me a bit; Annoy3: my teeth annoy me a lot;

Awake2: my teeth keep me awake a bit; Awake3: my teeth keep me awake a lot; Eat2: my teeth make it a bit hard to eat some foods; Eat3: my teeth make it really hard to eat some foods; Cry2: my teeth make me cry a bit; Cry3: my teeth make me cry a lot; LY: duration

Table S17: Adult and adolescent derived utilities for each health state defined by the CARIES-QC-U classification system

Health state	Adult value set	Adolescent value set	Health state	Adult value set	Adolescent value set
11111	1.000	1.015	22223	0.412	0.453
11112	1.000	0.937	22231	0.411	0.270
11113	0.942	0.946	22232	0.411	0.133
11121	0.942	0.861	22233	0.403	0.369
11122	0.903	0.903	22311	0.403	0.238
11123	0.903	0.814	22312	0.388	0.481
11131	0.901	0.876	22313	0.388	0.358
11132	0.901	0.785	22321	0.385	0.230
11133	0.877	0.877	22322	0.380	0.634
11211	0.845	0.823	22323	0.380	0.522
11212	0.845	0.727	22331	0.377	0.340
11213	0.843	0.795	22332	0.377	0.207
11221	0.843	0.696	22333	0.371	0.473
11222	0.836	0.891	23111	0.371	0.349
11223	0.836	0.801	23112	0.356	0.244
11231	0.827	0.836	23113	0.353	0.341
11232	0.827	0.741	23121	0.347	0.159
11233	0.819	0.796	23122	0.345	0.085
11311	0.804	0.745	23123	0.341	0.577
11312	0.804	0.642	23131	0.341	0.461
11313	0.780	0.746	23132	0.339	0.542
11321	0.778	0.715	23133	0.339	0.424
11322	0.769	0.751	23211	0.323	0.382
11323	0.769	0.649	23212	0.321	0.315
11331	0.746	0.652	23213	0.315	0.544
11332	0.746	0.542	23221	0.312	0.233
11333	0.739	0.762	23222	0.306	0.182

12111	0.739	0.660	23223	0.306	0.039
12112	0.739	0.782	23231	0.288	0.030
12113	0.739	0.682	23232	0.283	0.473
12121	0.737	0.731	23233	0.283	0.350
12122	0.737	0.627	23311	0.283	0.402
12123	0.730	0.699	23312	0.283	0.274
12131	0.730	0.593	23313	0.281	0.436
12132	0.728	0.667	23321	0.281	0.310
12133	0.728	0.558	23322	0.280	0.140
12211	0.722	0.654	23323	0.274	0.562
12212	0.720	0.621	23331	0.274	0.445
12213	0.713	0.733	23332	0.274	0.323
12221	0.707	0.860	23333	0.274	0.190
12222	0.707	0.767	31111	0.272	0.255
12223	0.704	0.669	31112	0.272	0.117
12231	0.681	0.563	31113	0.265	0.266
12232	0.681	0.693	31121	0.257	0.437
12233	0.681	0.586	31122	0.254	0.108
12311	0.672	0.604	31123	0.248	0.256
12312	0.672	0.490	31131	0.242	0.372
12313	0.670	0.570	31132	0.242	0.241
12321	0.670	0.453	31133	0.218	0.373
12322	0.664	0.686	31211	0.216	0.334
12323	0.664	0.578	31212	0.215	0.204
12331	0.649	0.777	31213	0.215	0.062
12332	0.649	0.677	31221	0.184	0.255
12333	0.646	0.571	31222	0.184	0.117
13111	0.642	0.639	31223	0.183	-0.067
13112	0.642	0.527	31231	0.178	0.394
13113	0.640	0.582	31232	0.178	0.265
13121	0.640	0.466	31233	0.177	0.420

13122	0.631	0.510	31311	0.177	0.293
13123	0.631	0.389	31312	0.175	0.355
13131	0.623	0.458	31313	0.175	0.223
13132	0.616	0.583	31321	0.160	0.257
13133	0.616	0.606	31322	0.160	0.177
13211	0.614	0.548	31323	0.158	0.215
13212	0.608	0.695	31331	0.151	0.356
13213	0.608	0.588	31332	0.151	0.090
13221	0.607	0.512	31333	0.149	0.014
13222	0.605	0.475	32111	0.145	0.521
13223	0.584	0.696	32112	0.145	0.401
13231	0.584	0.539	32113	0.119	0.144
13232	0.584	0.420	32121	0.119	0.306
13233	0.575	0.624	32122	0.119	0.171
13311	0.575	0.511	32123	0.110	0.113
13312	0.573	0.402	32131	0.110	-0.034
13313	0.573	0.273	32132	0.092	-0.043
13321	0.567	0.531	32133	0.087	0.414
13322	0.567	0.411	32211	0.087	0.286
13323	0.566	0.555	32212	0.080	0.238
13331	0.566	0.437	32213	0.080	0.099
13332	0.564	0.495	32221	0.078	0.166
13333	0.564	0.372	32222	0.078	0.023
21111	0.558	0.504	32223	0.061	0.014
21112	0.550	0.599	32231	0.055	0.168
21113	0.550	0.485	32232	0.054	0.197
21121	0.549	0.403	32233	0.052	0.125
21122	0.547	0.365	32311	0.046	0.309
21123	0.544	0.713	32312	0.046	0.174
21131	0.544	0.607	32313	0.022	0.311
21132	0.541	0.496	32321	0.022	0.113

21133	0.535	0.648	32322	0.022	-0.033
21211	0.535	0.537	32323	0.014	0.220
21212	0.526	0.601	32331	0.014	0.079
21213	0.519	0.443	32332	-0.004	0.071
21221	0.517	0.378	32333	-0.012	0.189
21222	0.508	0.298	33111	-0.012	0.046
21223	0.508	0.449	33112	-0.013	-0.143
21231	0.508	0.324	33113	-0.018	0.332
21232	0.485	0.507	33121	-0.018	0.198
21233	0.478	0.462	33122	-0.036	0.190
21311	0.478	0.338	33123	-0.043	-0.005
21312	0.476	0.549	33131	-0.045	-0.084
21313	0.476	0.430	33132	-0.077	0.074
21321	0.470	0.386	33133	-0.083	0.019
21322	0.470	0.256	33211	-0.083	-0.133
21323	0.467	0.319	33212	-0.101	-0.142
21331	0.467	0.185	33213	-0.109	-0.025
21332	0.461	0.330	33221	-0.115	0.171
21333	0.452	0.426	33222	-0.115	0.028
22111	0.450	0.177	33223	-0.118	0.097
22112	0.446	0.585	33231	-0.118	-0.050
22113	0.446	0.469	33232	-0.135	-0.059
22121	0.444	0.526	33233	-0.141	0.099
22122	0.444	0.406	33311	-0.174	0.042
22123	0.444	0.321	33312	-0.174	-0.108
22131	0.443	0.348	33313	-0.206	-0.246
22132	0.441	0.281	33321	-0.238	-0.079
22133	0.438	0.728	33322	-0.240	-0.160
22211	0.438	0.623	33323	-0.279	-0.054
22212	0.435	0.452	33331	-0.279	-0.210
22213	0.435	0.327	33332	-0.297	-0.220

22221	0.427	0.398	33333	-0.402	-0.326
22222	0.421	0.527			

Notes: * The utility for this health state was predicted to be above 1, so has been capped at 1.
